# Supplementary material for: Introducing ACEs (Adverse Childhood Experiences) and Resilience to First-Year Medical Students
Source: MedEdPORTAL. 2020 Sep 15;16:10964. doi: 10.15766/mep_2374-8265.10964 (PMC7499813; doi:10.15766/mep_2374-8265.10964)
Supplement: Supplementary file 1 — The Case of Ms. Anthony.docxIntroducing ACEs Presentation.pptxSelf-Assessment.docx [file mep_2374-8265.10964-s001.zip › A. The Case of Ms. Anthony.docx]

**The Case of Ms Anthony**

This is the case of Ms. Anthony, a highly skilled professional, a nurse, who was referred by the head of Orthopedics (Dr. Campbell) to the head of Psychiatry (Dr. Yudofsky).

**The Referral**

Dr. Campbell phones Dr. Yudofsky and says, “Good morning, Stuart. I hope I’m not interrupting you, but I need you to see a patient ASAP. It’s Ms. Anita Anthony, one of the best surgery nurses we have here. It got to the point that our place can’t run without her, and she’s been missing a lot of work lately. Although she has plenty of troubles with her body, I think most of her problems are in her head. She vehemently disagrees. Try to fit her in today, if you can. I gotta run now. You’re going to love Anita!”

**The first Meeting – Going Beneath the Surface**

The first meeting between Dr. Yudofsky and Ms. Anthony takes place. Ms. Anthony begins by saying, “This will never work.” “Why?” Dr. Yudofsky asks. Ms. Anthony replies “Well, just look at you and look at me. We have absolutely nothing in common. I see a skinny little white man in a fancy office that is set up to do a lot of talking. Unless you’re blind, you see a big – a too big – black woman.” Dr. Yudofsky replies “That’s not what I see, Ms. Anthony. I see a person about whom I have a great deal to learn. On the surface you may be big and black, and I may be little and white, but that’s just the surface. On the surface, surgery, where you work, cuts and sews, and psychiatry, where I work, talks and talks. But that’s just the surface. The fact is that the two specialties have a lot in common.” “Oh, here we go! According to you psychiatrists, everybody is misunderstood. Next thing you’ll be telling me that the reason I’m 100 pounds overweight is because my mother misunderstood me when I was in diapers!” Ms. Anthony retorts. Dr. Yudofsky responds evenly, “Most people think surgeons work with their hands and psychiatrists work with their mouths. It couldn’t be further from the truth. We both work with our minds, and we both do our work deep beneath the surface. As you undoubtedly know, one should never judge the quality of a heart transplant by how the skin incision looks. Nor would I ever presume to know anything about you from the color of your skin or how much you weigh. Let’s start by your telling me why you came by to see me today.”

**Establishing the Chief Complaint**

Ms. Anthony replies, “The only reason I came here is because my doctor told me I had to come. I’m eating myself to death. I have “high blood” everything: high blood sugar, high blood cholesterol, and high blood pressure. On top of that I have arthritis in both my knees that’s so bad I can hardly walk. It all started when about ten years ago when I fell and fractured both of my kneecaps. And I *don’t* need you to tell me that my weight doesn’t help anything. What I *do* need is to get two knee replacements, but Dr. Campbell won’t touch me unless he gets the green light from you. That’s all I need from you.” Ms. Anthony did not return to the clinic.

One year later, Dr. Yudofsky is called to the ER because a patient with a suicide attempt has refused to speak to any doctor, except Dr. Yudofsky. Her name is Anita Anthony. After the ER visit, Ms. Anthony begins to attend appointments regularly with Dr. Yudofsky. Dr. Yudofsky learns about her background. Her father was a bright man who graduated with highest honors in chemical engineering. He married his high school sweetheart and joined the US Marines. Ms. Anthony’s mother was a pediatric nurse in a city hospital. Kyle, her brother was two years older. Ms. Anthony describes her mother as “physically beautiful, passive, distant, and for the most part, incompetent.”

**Life Transforming Event**

Ms. Anthony reports a life-transforming event. She recounts, “Three soldiers came to our little house to tell our mother that Daddy had been killed in Desert Storm. After this, mother suffered a nervous breakdown. All she did was lie around in bed most of the day or go to church for endless hours. We did everything for ourselves. After Daddy died, we essentially raised ourselves.” Three months later, Ms. Anthony’s mother introduced Mr. Neil, a retired policeman, to the children. Soon after, he was living in their home. Mr. Neil was “on disability for something.” He often came home smelling of alcohol, and “At night, I rarely saw him without a beer in his hand. He was the exact opposite of my father. I have often thought that while my father was *killed* in action, my mother was *missing* in action. Kyle filled in the void for me after Daddy was gone.” Kyle went off to college, earned a Ph.D., joined the army, and was deployed to both Iraq and Afghanistan.”

Several months into treatment, Ms. Anthony confronts Dr. Yudofsky and says, “The reason I came to see you in the first place was for you to put me on a diet. I weigh the same as I always have. You’ve been bugging me for over a year about practically everything else. So how come you never ask me about my weight?” “We don’t understand why you gained so much weight in the first place, and without that understanding, your going on a diet would be doomed to failure” Dr. Yudofsky replies. To this, Ms. Anthony responds “I told you I began to gain weight when I started eating a lot of junk food when I was 16. I eat it compulsively. That’s the true and only cause.” “If you know the true and only cause, why can’t you stop eating junk food?” Dr. Yudofsky asks. “You’re the famous doctor; you tell me!” says Ms. Anthony.

**The Person prior to the Trauma**

With Ms. Anthony’s permission, Dr. Yudofsky obtains photos from her high school yearbook around the time she began gaining weight. At their next appointment, he shows this to Ms. Anthony. For a moment, Ms. Anthony stares at the photograph. She is mute and immobile. Then she begins to tremble, then wail, then quake volcanically. She makes several visible attempts to stop bawling, but wave after wave of emotions continues to crash through. Finally, she says haltingly “I’m sorry for behaving like an idiot. I have no idea what came over me.” “Pent-up pressure of something that you have been trying to bury must have burst to the surface,” Dr. Yudofsky says. “What haven’t you been able to talk about, Ms. Anthony? I believe you know.”

**Disclosing the Abuse**

Over the next hour, Ms. Anthony tells Dr. Yudofsky that she began to gain weight soon after becoming pregnant at age 16. She and Kyle had grown up sharing the only other bedroom in their small house. Shortly after Kyle departed for college, Neil started sneaking into her room late at night to molest her. She told her mother, who never did anything to stop him. She thought about telling Kyle, but he had just started in the military academy, and she didn’t want to derail his path in life.

**Defending the Post-Trauma Self**

She became horribly uncomfortable with her body, felt weak and vulnerable, a feeling she hated because she feared she was becoming her mother. She also felt others could see through her to her trauma. Ms. Anthony says gaining weight in high school “made me feel more comfortable.” She recounts, “When I graduated from high school, I was over 6 feet tall and weighed 225 pounds. You can bet your boots that nobody was going to mess with me! And, as you know, Dr. Yudofsky, I’m not afraid to come on strong. In school and later at work, I also had my act together. I don’t like being attacked for not doing my job.”

**The Cost of this Defense**

Dr. Yudofsky observes, “There is no question that you are an impressive and imposing woman, Ms. Anthony. But your resolution has come at a considerable cost. You now weigh 275 pounds, which is making you physically vulnerable. Besides, both your weight and your aggressive personality push away many people who are not at all threatening to you.”

**One Year Later**

One year later, after Ms. Anthony attended sessions regularly, she has made significant progress. After not having danced in 20 years, Ms. Anthony has rediscovered how accomplished she is at dancing. She is now taking dancing classes and has been asked out on several dates. Her insight and self-confidence have grown; she has changed and is changing her behavior.

**The Courage Required for Psychological vs. Physical Treatment**

Ms. Anthony’s final comments were, “I’ve lost almost half of my body weight in a year. Don’t you find it interesting that I find the psychotherapy part so much more challenging than the dieting part?” to which Dr. Yudofsky replied, “There’s a difference?”
