# Supplementary material for: Introducing ACEs (Adverse Childhood Experiences) and Resilience to First-Year Medical Students
Source: MedEdPORTAL. 2020 Sep 15;16:10964. doi: 10.15766/mep_2374-8265.10964 (PMC7499813; doi:10.15766/mep_2374-8265.10964)
Supplement: Supplementary file 1 — The Case of Ms. Anthony.docxIntroducing ACEs Presentation.pptxSelf-Assessment.docx [file mep_2374-8265.10964-s001.zip › B. Introducing ACEs Presentation.pptx]

## Slide 1
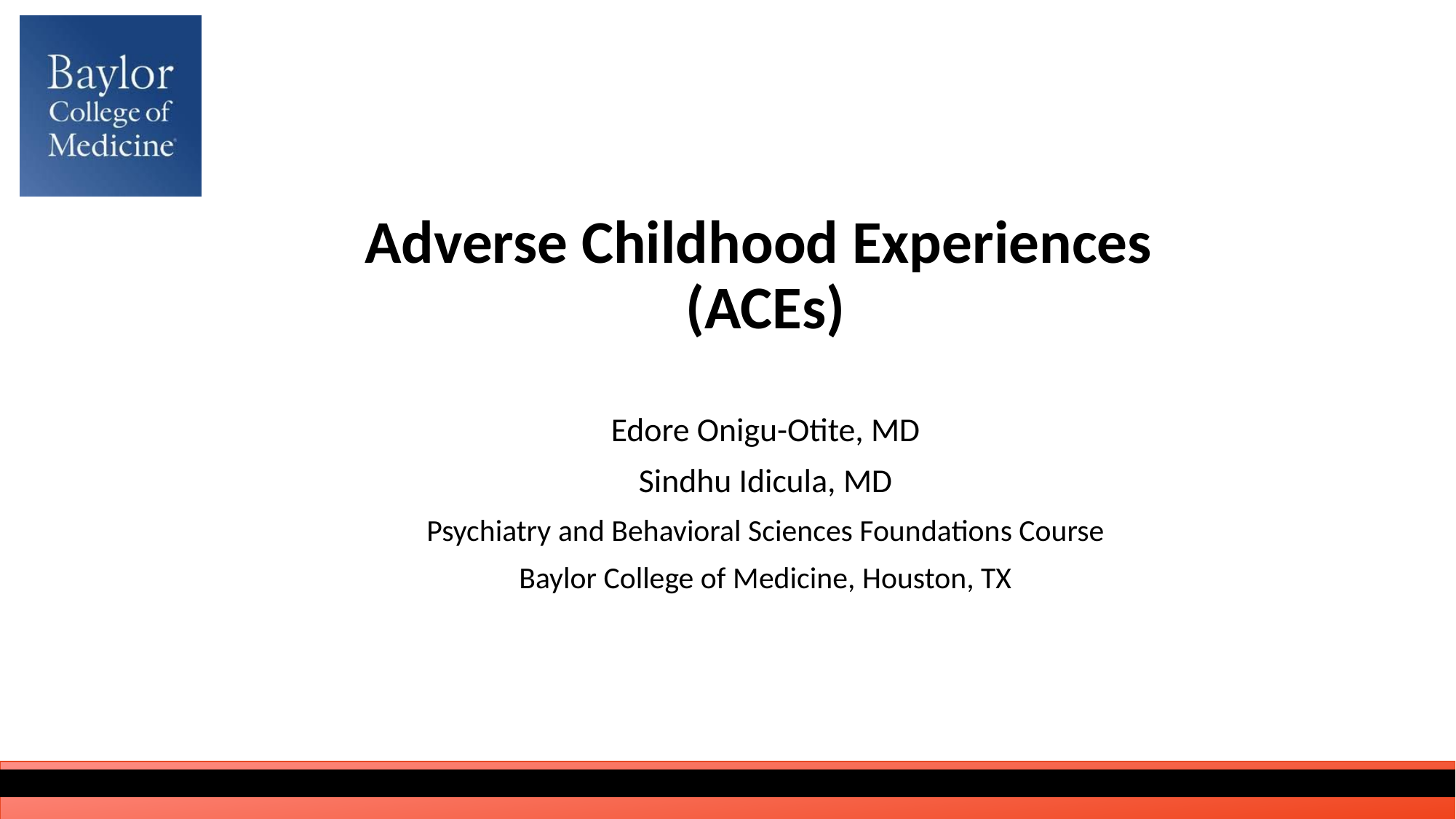

# Adverse Childhood Experiences (ACEs)
Edore Onigu-Otite, MD
Sindhu Idicula, MD
Psychiatry and Behavioral Sciences Foundations Course
Baylor College of Medicine, Houston, TX
1

## Slide 2
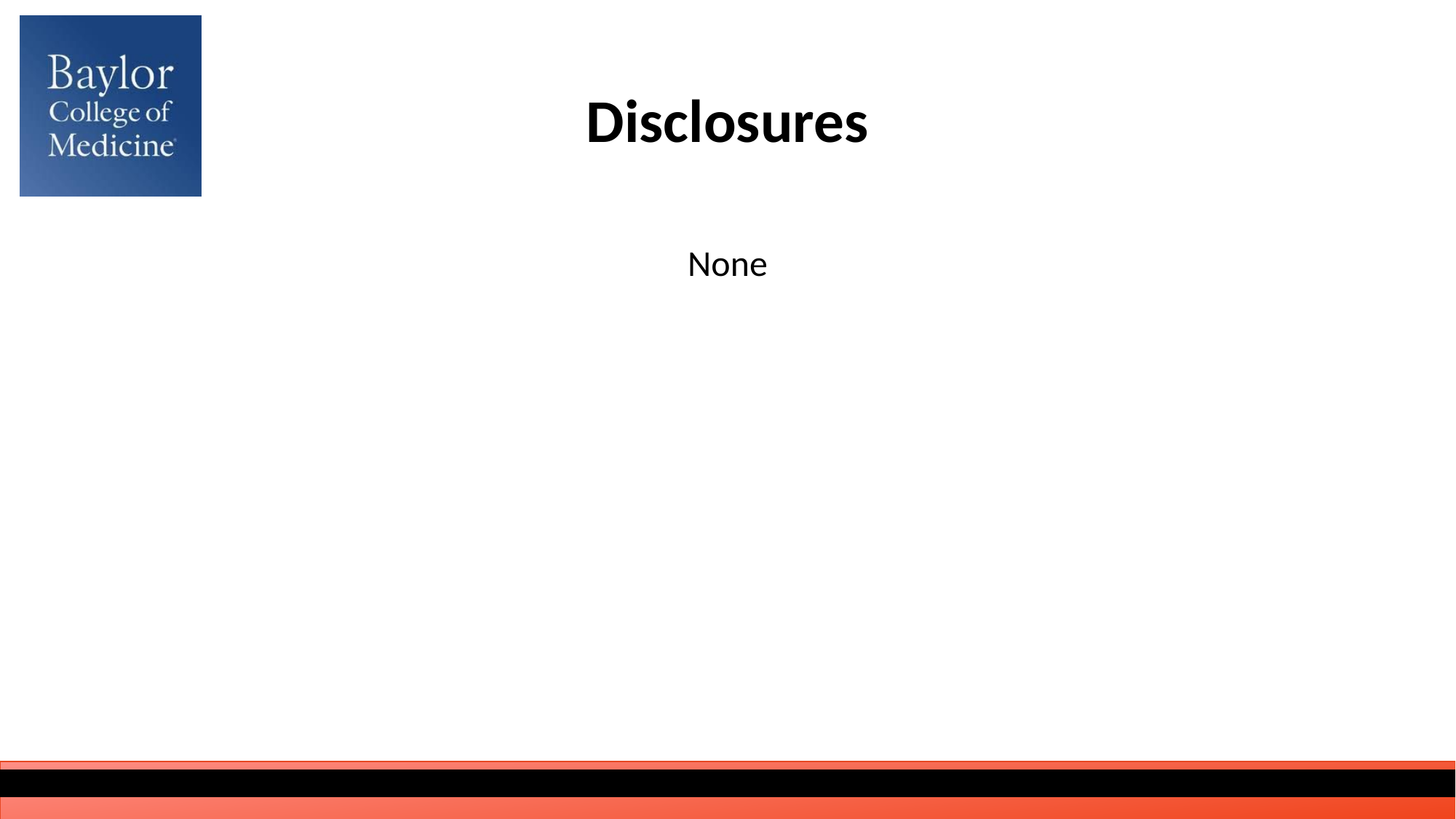

# Disclosures
None
2

## Slide 3
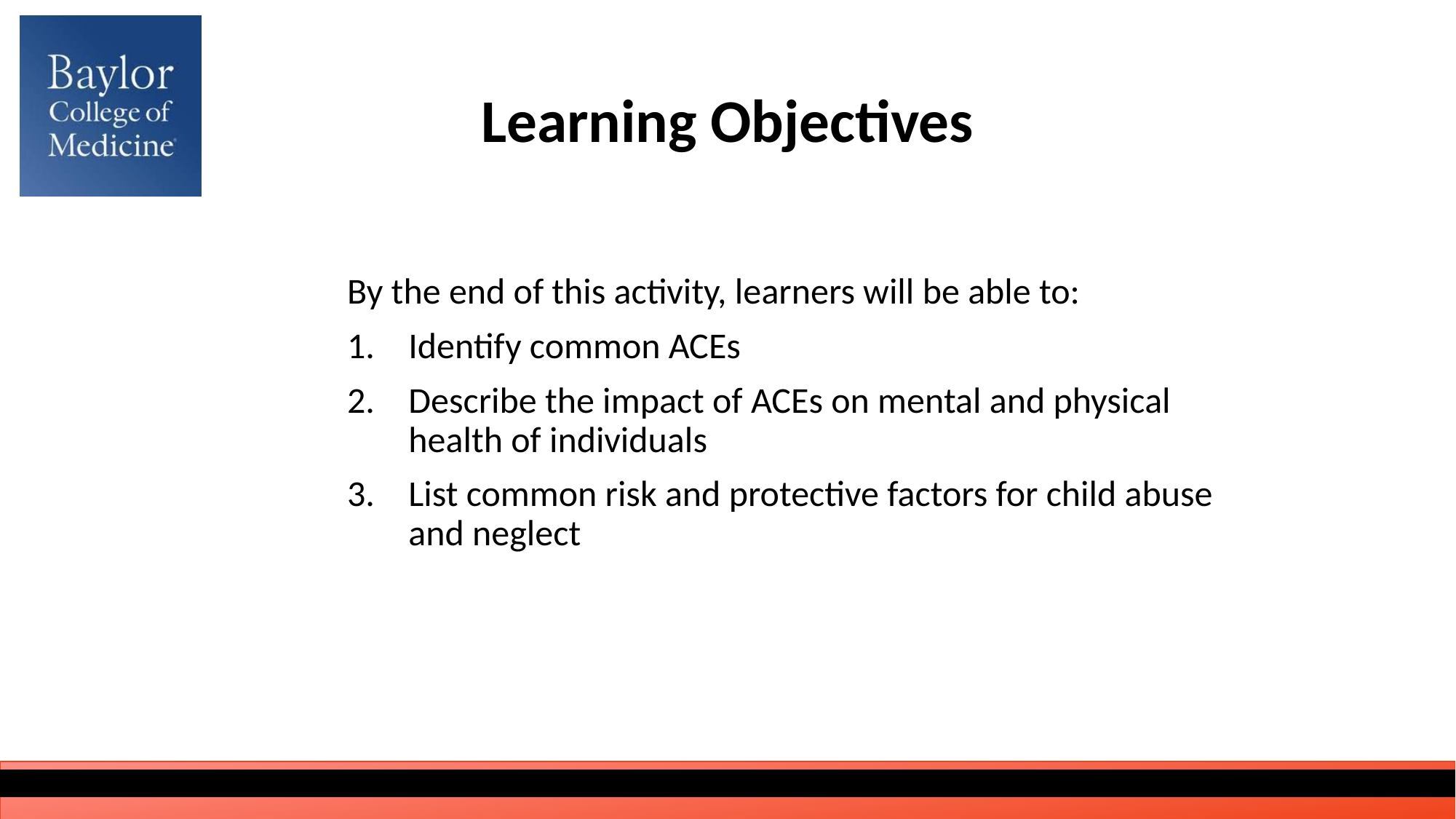

# Learning Objectives
By the end of this activity, learners will be able to:
Identify common ACEs
Describe the impact of ACEs on mental and physical health of individuals
List common risk and protective factors for child abuse and neglect
3

## Slide 4
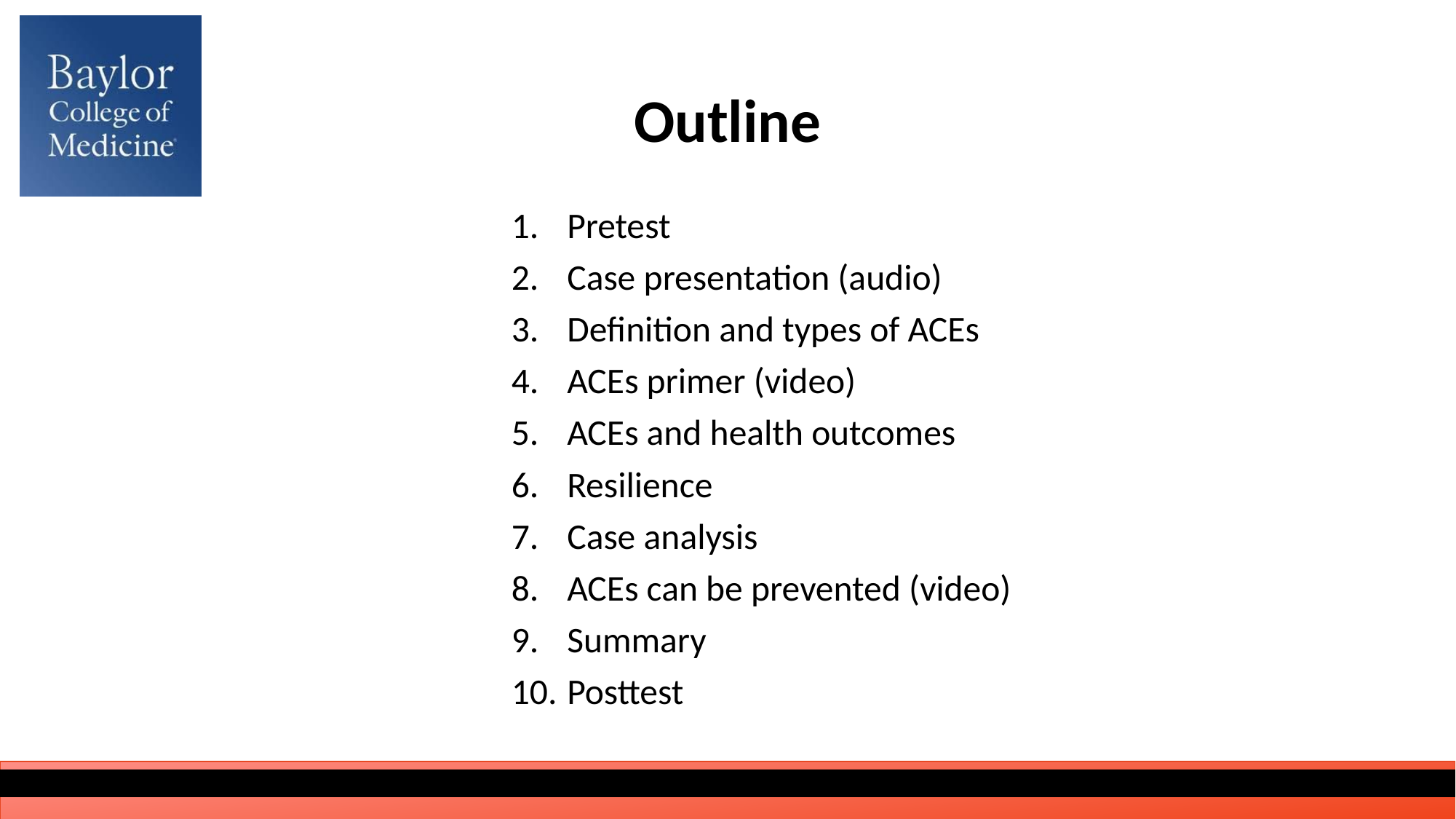

# Outline
Pretest
Case presentation (audio)
Definition and types of ACEs
ACEs primer (video)
ACEs and health outcomes
Resilience
Case analysis
ACEs can be prevented (video)
Summary
Posttest
4

## Slide 5
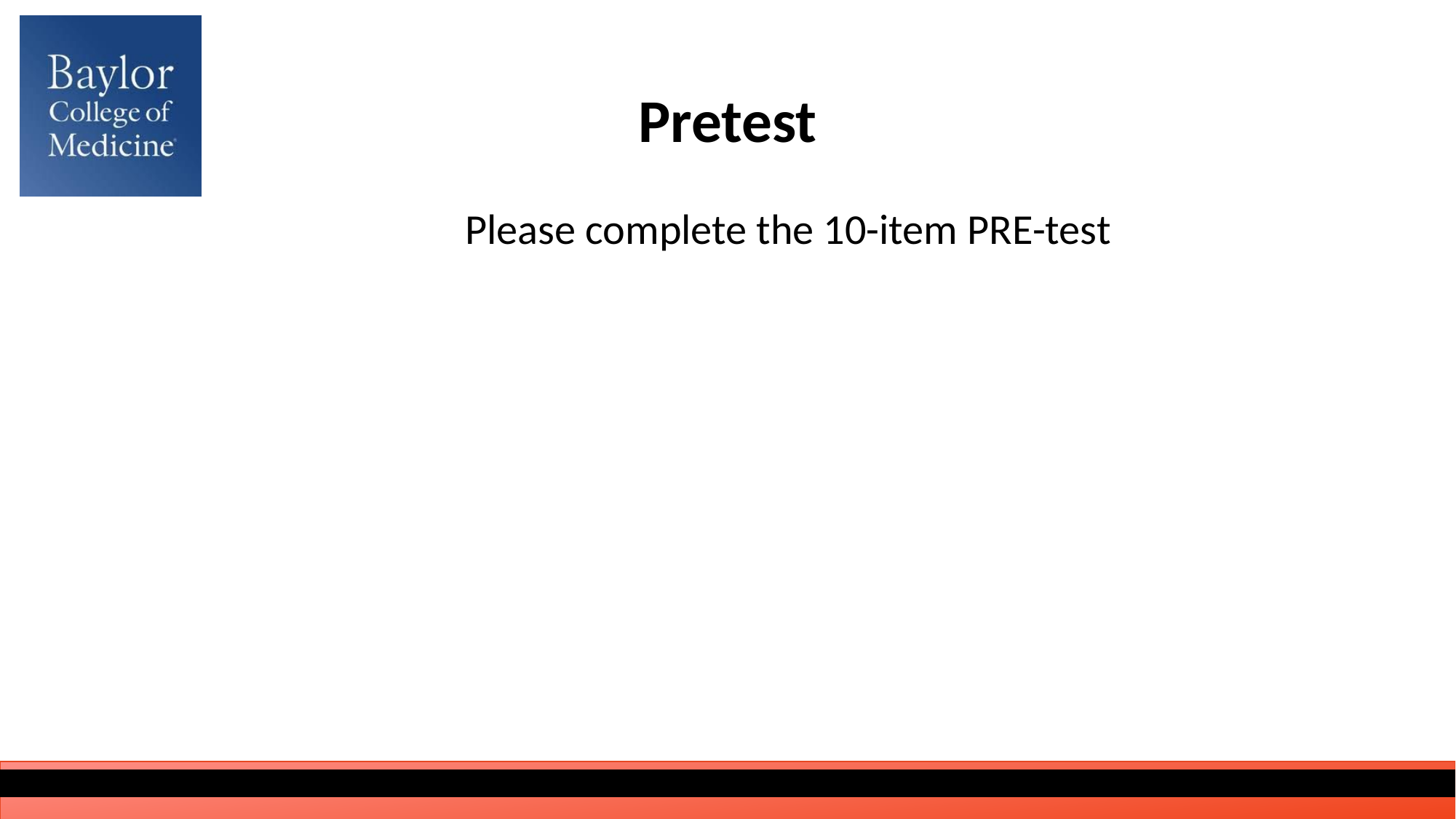

# Pretest
Please complete the 10-item PRE-test
5

## Slide 6
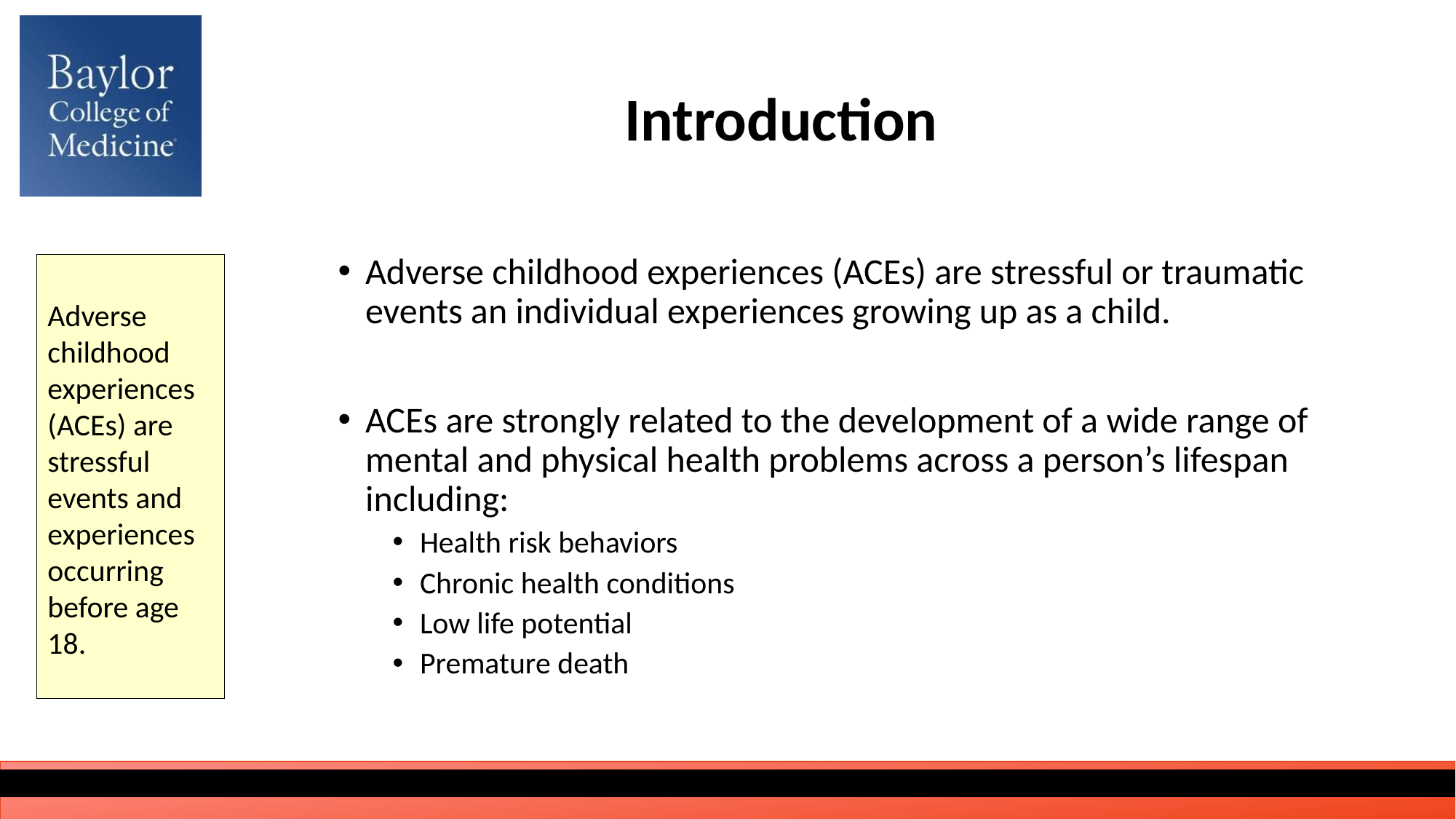

# Introduction
Adverse childhood experiences (ACEs) are stressful or traumatic events an individual experiences growing up as a child.
ACEs are strongly related to the development of a wide range of mental and physical health problems across a person’s lifespan including:
Health risk behaviors
Chronic health conditions
Low life potential
Premature death
Adverse childhood experiences (ACEs) are stressful events and experiences occurring before age 18.
6

## Slide 7
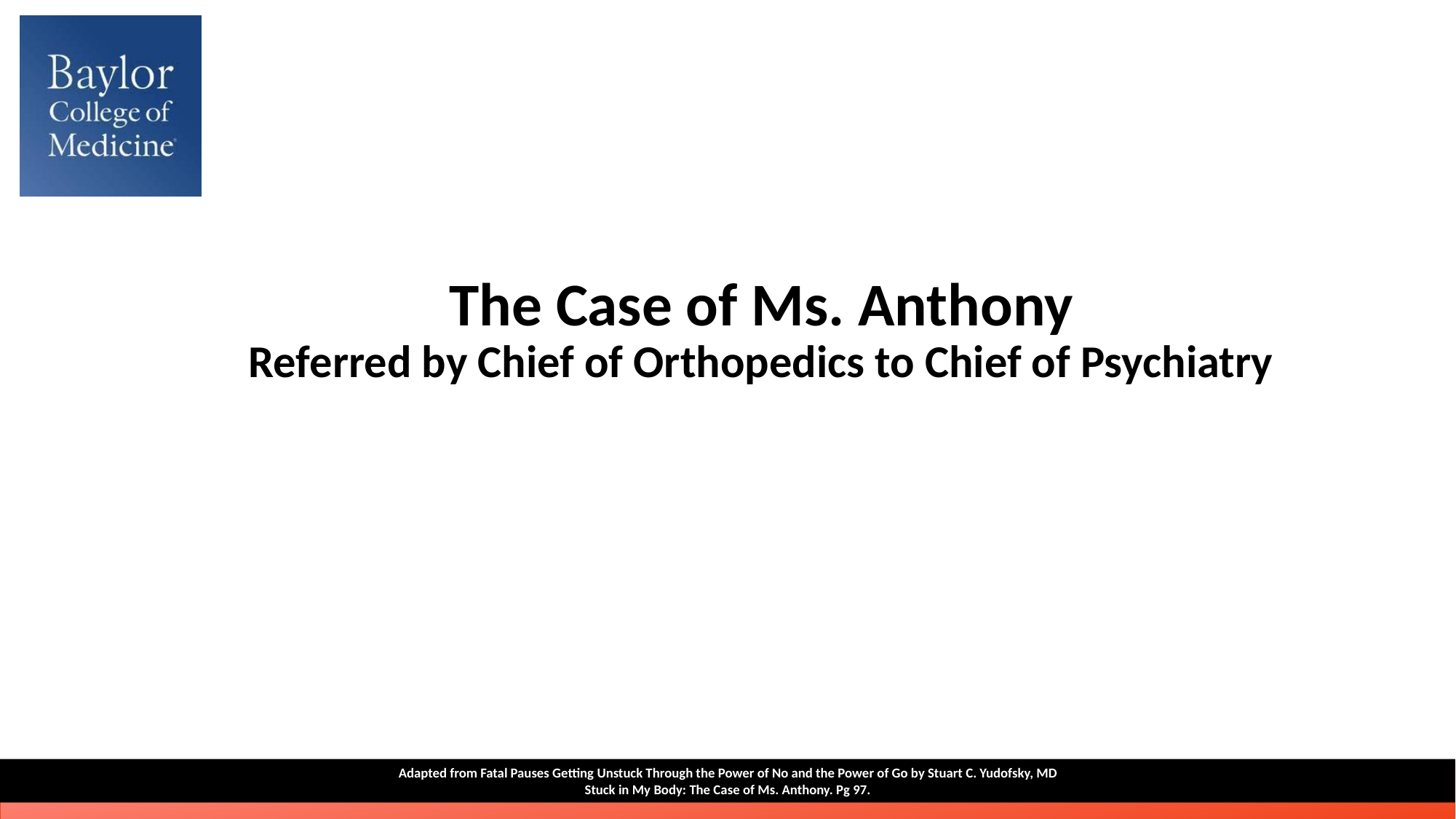

# The Case of Ms. Anthony Referred by Chief of Orthopedics to Chief of Psychiatry
Adapted from Fatal Pauses Getting Unstuck Through the Power of No and the Power of Go by Stuart C. Yudofsky, MD
Stuck in My Body: The Case of Ms. Anthony. Pg 97.
7

## Slide 8
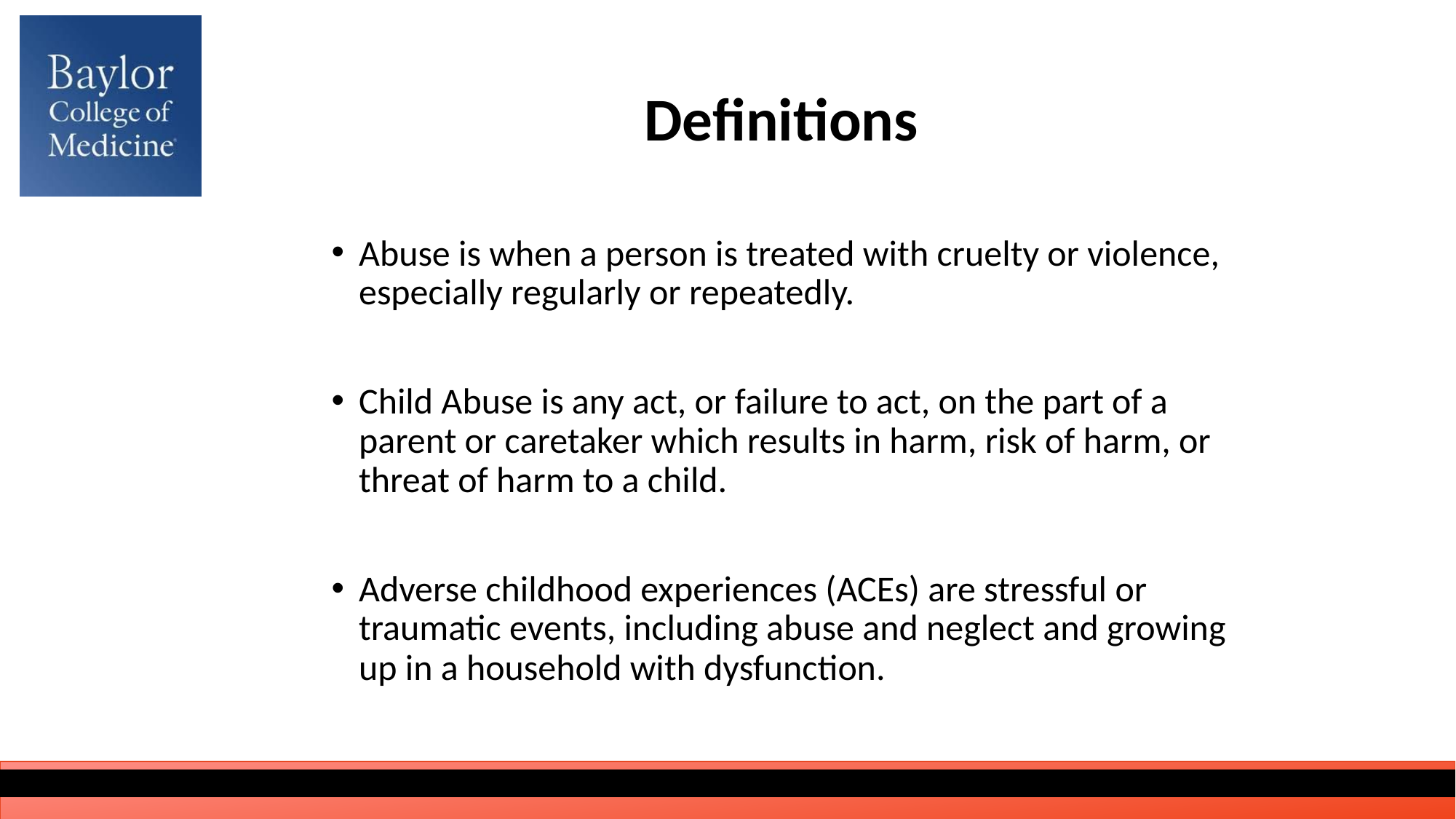

# Definitions
Abuse is when a person is treated with cruelty or violence, especially regularly or repeatedly.
Child Abuse is any act, or failure to act, on the part of a parent or caretaker which results in harm, risk of harm, or threat of harm to a child.
Adverse childhood experiences (ACEs) are stressful or traumatic events, including abuse and neglect and growing up in a household with dysfunction.
8

## Slide 9
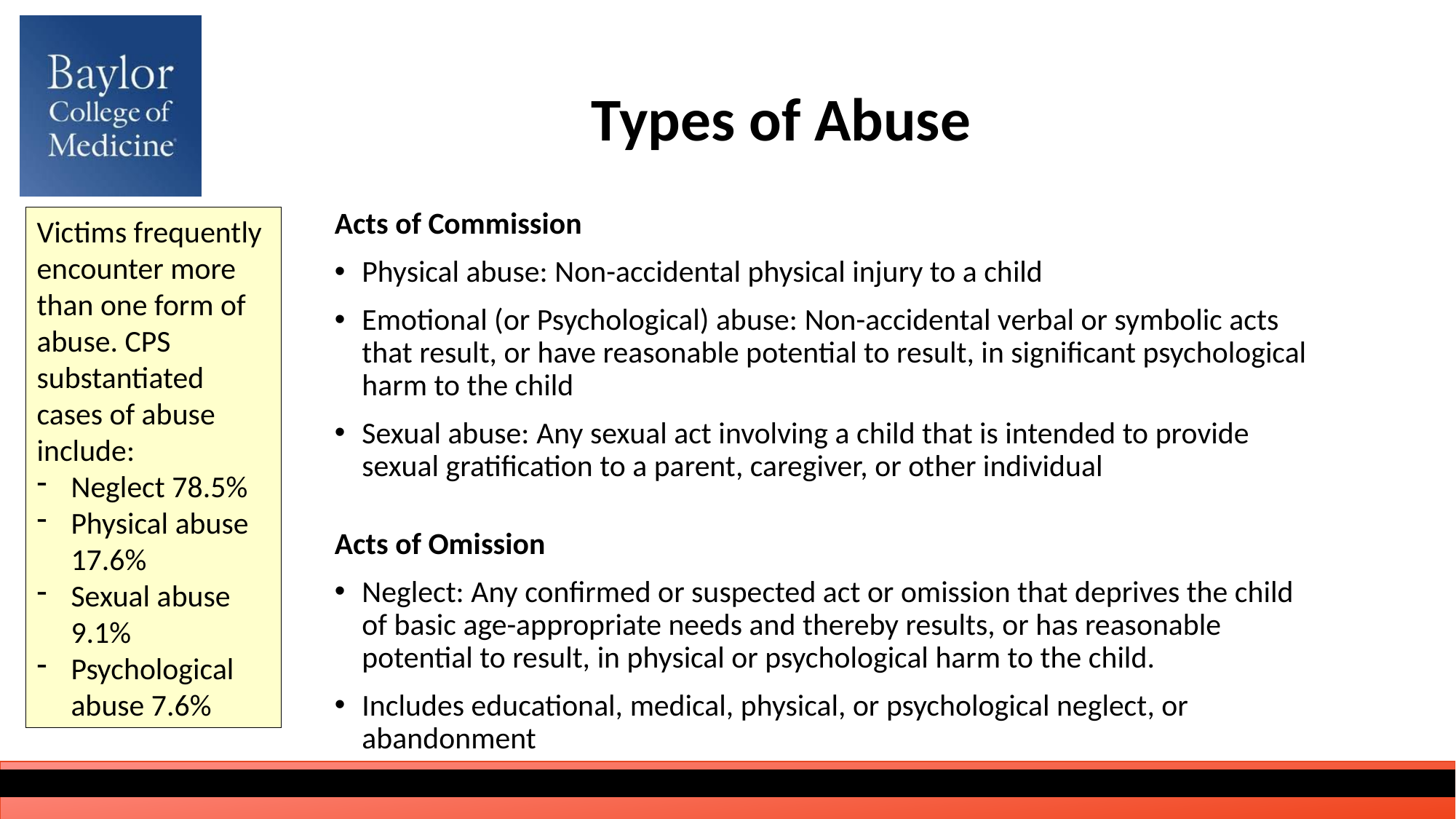

# Types of Abuse
Acts of Commission
Physical abuse: Non-accidental physical injury to a child
Emotional (or Psychological) abuse: Non-accidental verbal or symbolic acts that result, or have reasonable potential to result, in significant psychological harm to the child
Sexual abuse: Any sexual act involving a child that is intended to provide sexual gratification to a parent, caregiver, or other individual
Acts of Omission
Neglect: Any confirmed or suspected act or omission that deprives the child of basic age-appropriate needs and thereby results, or has reasonable potential to result, in physical or psychological harm to the child.
Includes educational, medical, physical, or psychological neglect, or abandonment
Victims frequently encounter more than one form of abuse. CPS substantiated cases of abuse include:
Neglect 78.5%
Physical abuse 17.6%
Sexual abuse 9.1%
Psychological abuse 7.6%
9

## Slide 10
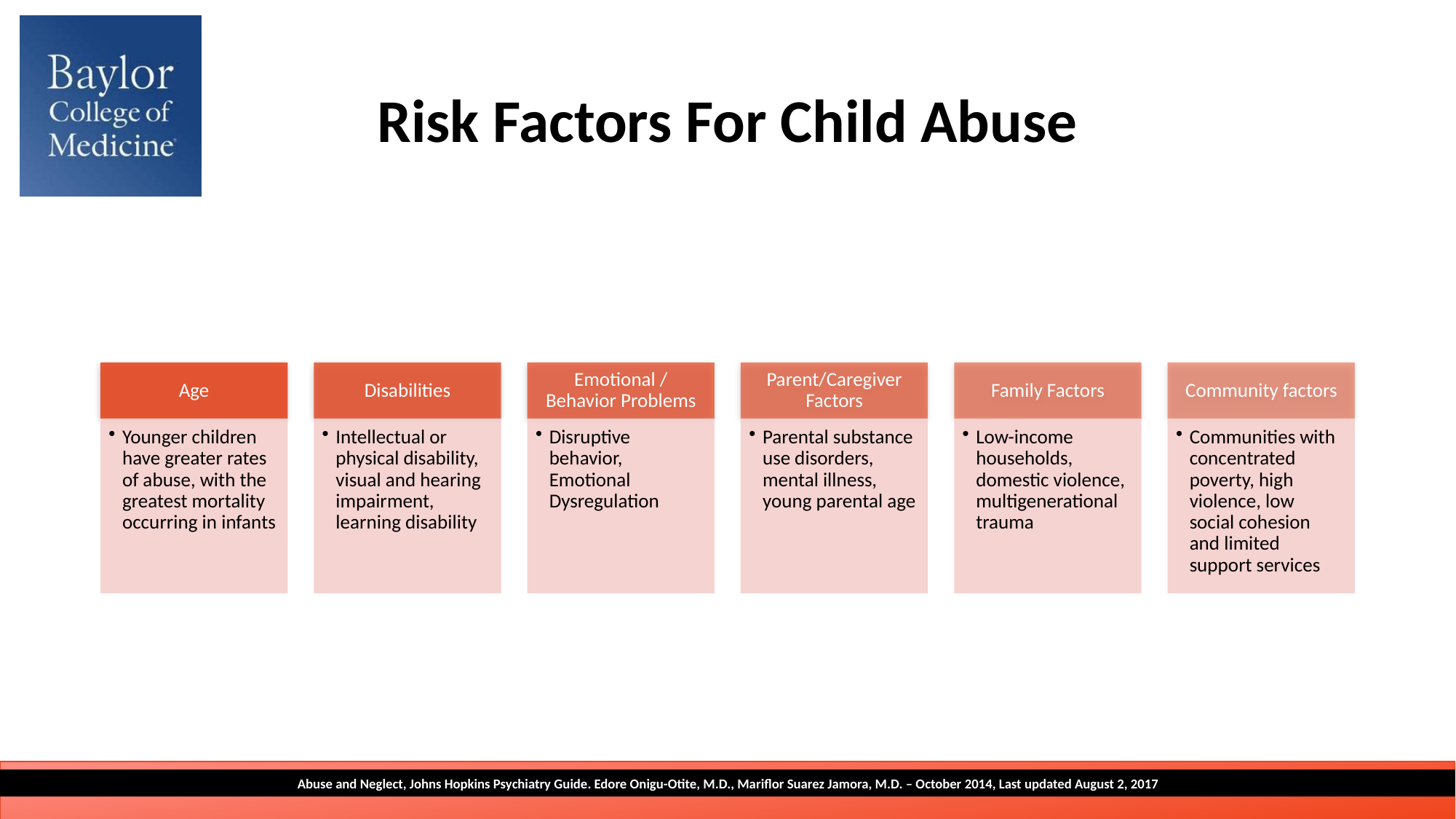

# Risk Factors For Child Abuse
10
Abuse and Neglect, Johns Hopkins Psychiatry Guide. Edore Onigu-Otite, M.D., Mariflor Suarez Jamora, M.D. – October 2014, Last updated August 2, 2017

## Slide 11
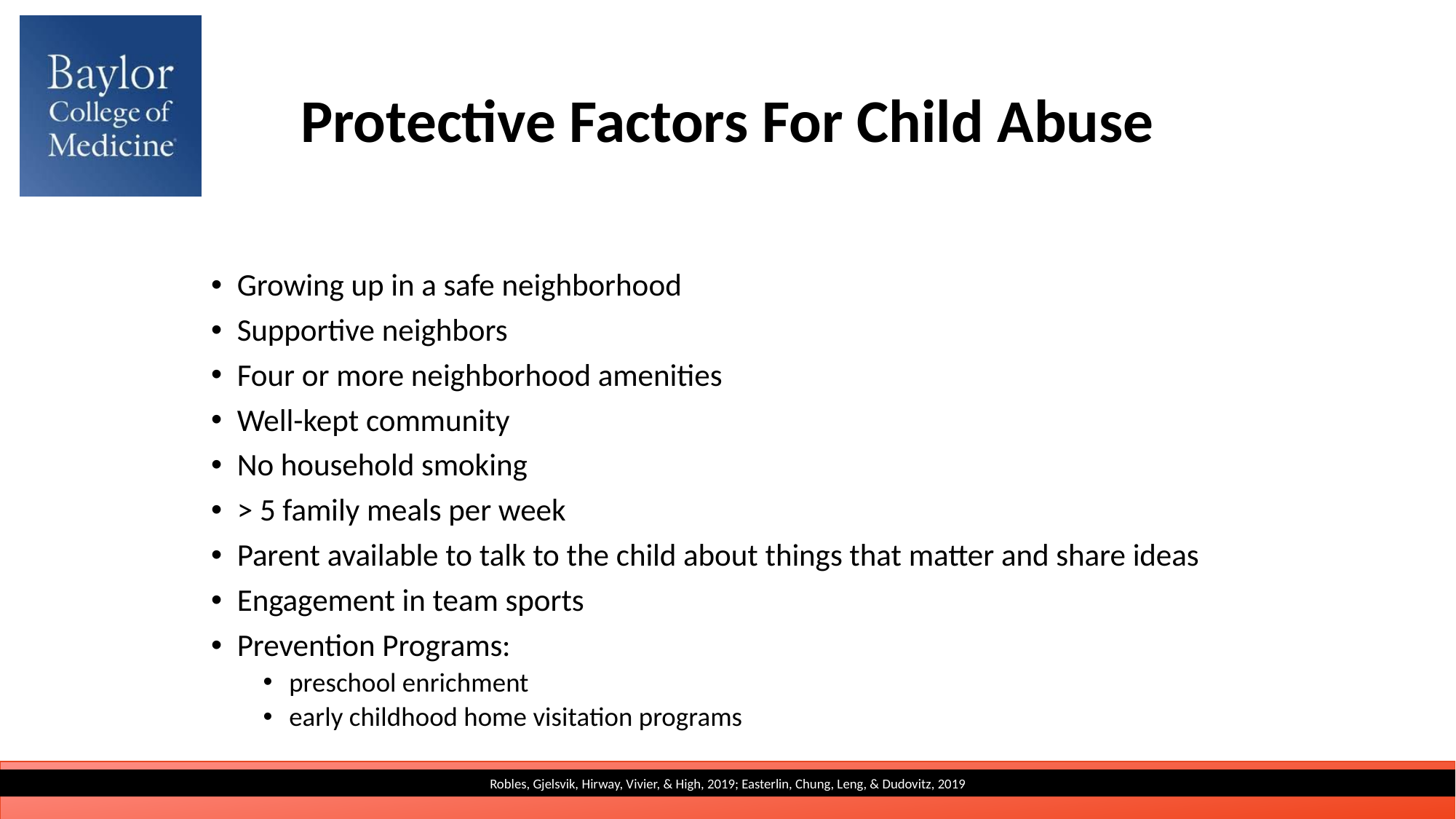

# Protective Factors For Child Abuse
Growing up in a safe neighborhood
Supportive neighbors
Four or more neighborhood amenities
Well-kept community
No household smoking
> 5 family meals per week
Parent available to talk to the child about things that matter and share ideas
Engagement in team sports
Prevention Programs:
preschool enrichment
early childhood home visitation programs
11
Robles, Gjelsvik, Hirway, Vivier, & High, 2019; Easterlin, Chung, Leng, & Dudovitz, 2019

## Slide 12
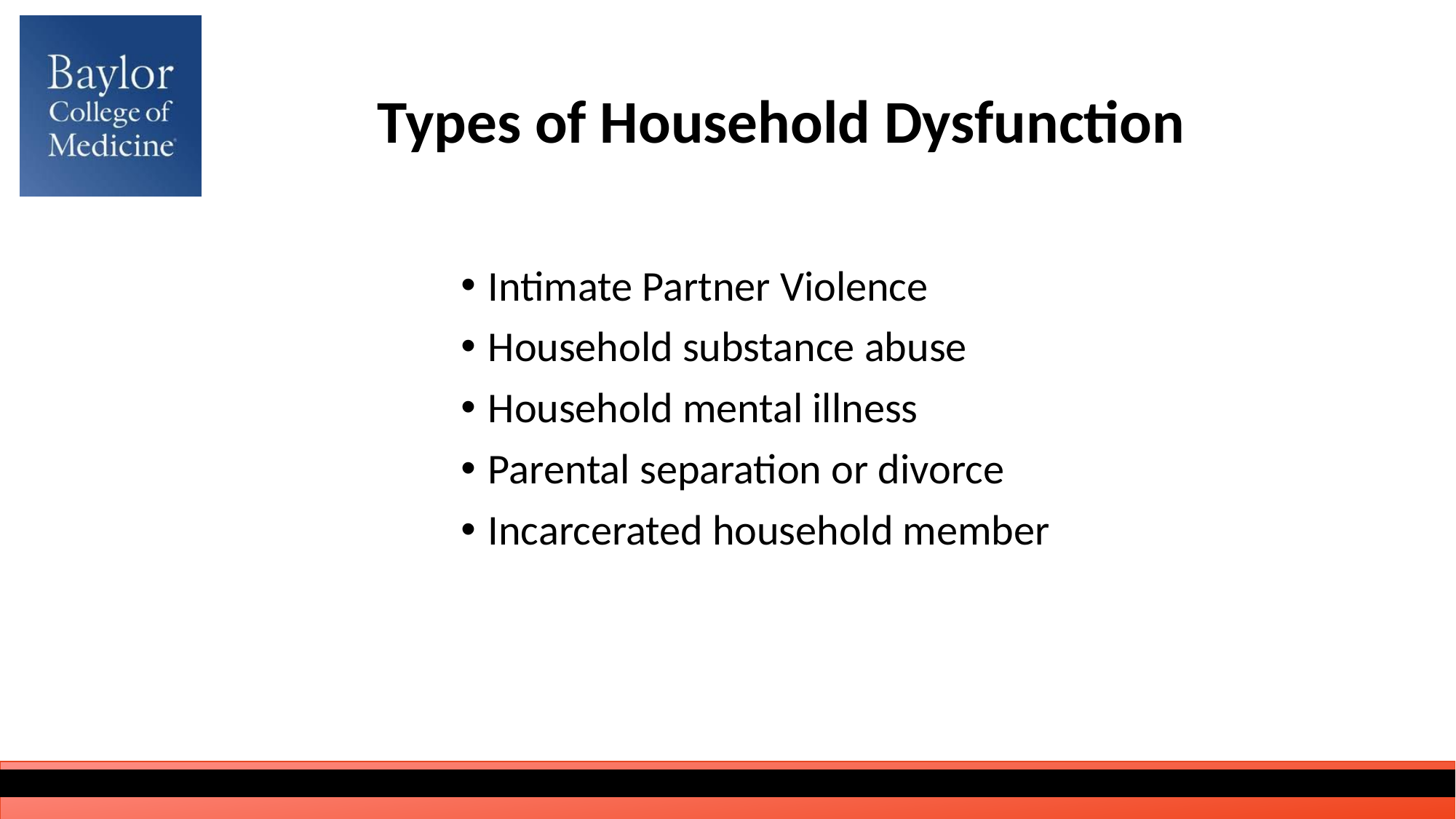

# Types of Household Dysfunction
Intimate Partner Violence
Household substance abuse
Household mental illness
Parental separation or divorce
Incarcerated household member
12

## Slide 13
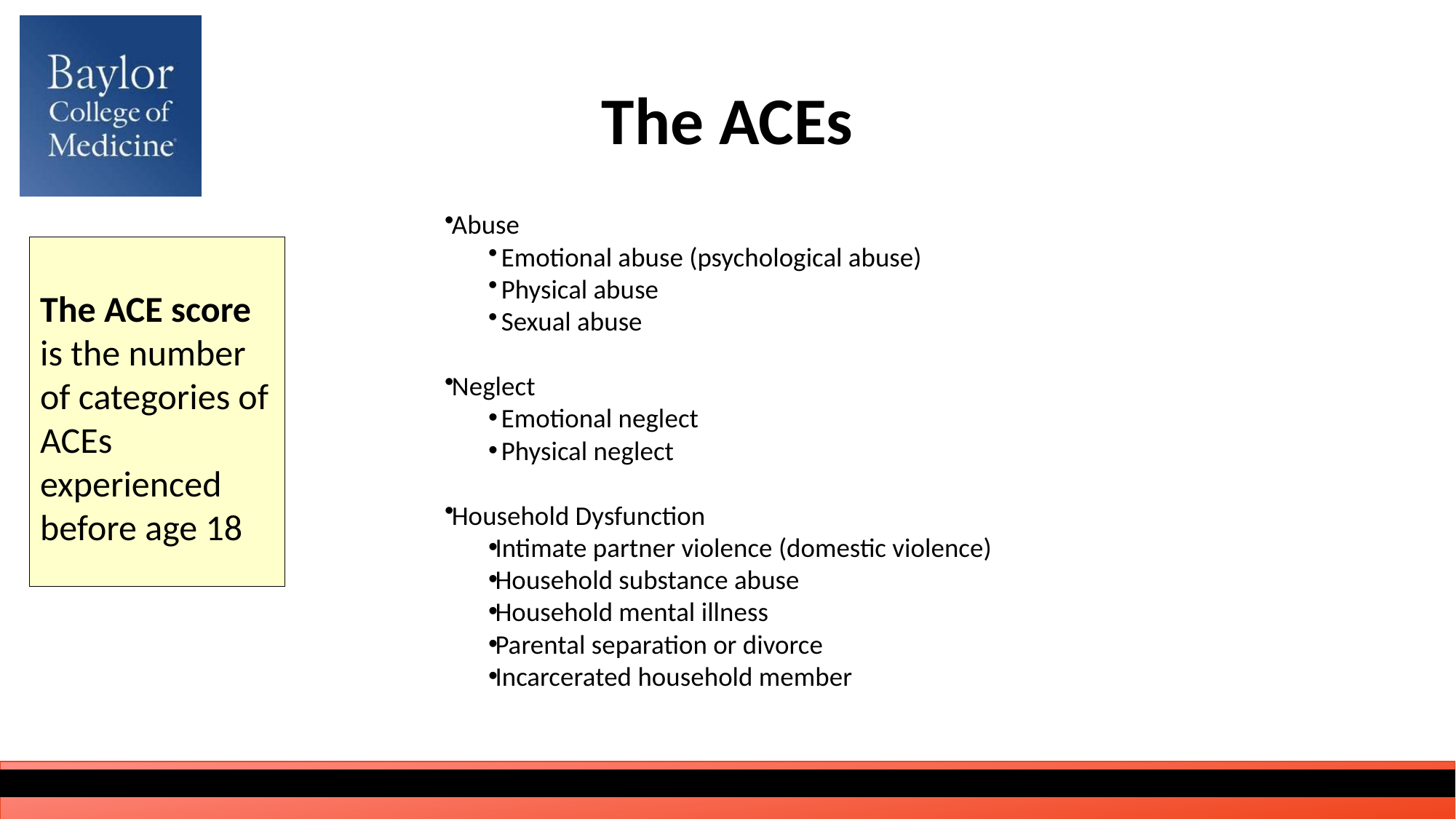

# The ACEs
Abuse
 Emotional abuse (psychological abuse)
 Physical abuse
 Sexual abuse
Neglect
 Emotional neglect
 Physical neglect
Household Dysfunction
Intimate partner violence (domestic violence)
Household substance abuse
Household mental illness
Parental separation or divorce
Incarcerated household member
The ACE score
is the number of categories of ACEs experienced before age 18
13

## Slide 14
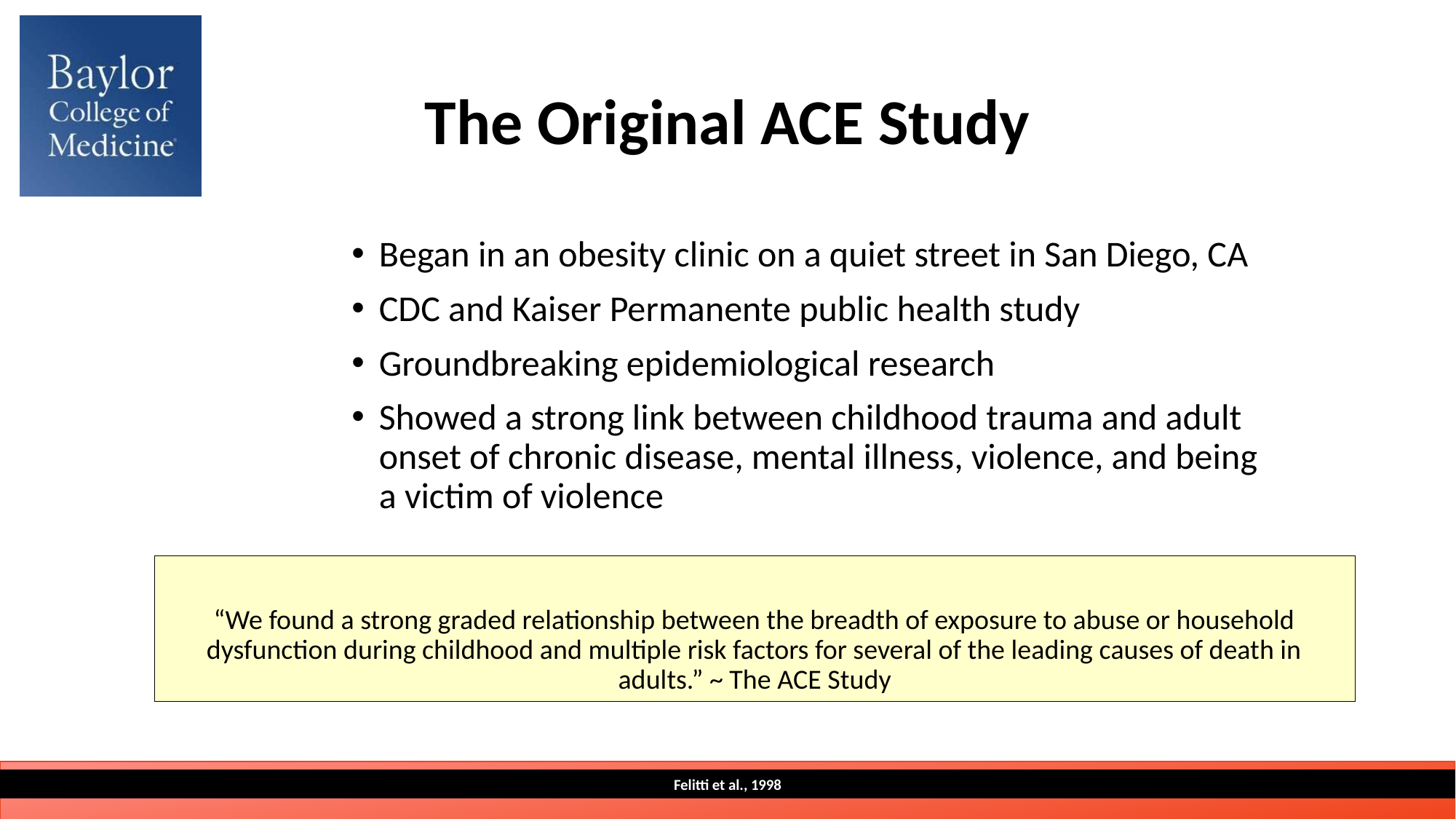

# The Original ACE Study
Began in an obesity clinic on a quiet street in San Diego, CA
CDC and Kaiser Permanente public health study
Groundbreaking epidemiological research
Showed a strong link between childhood trauma and adult onset of chronic disease, mental illness, violence, and being a victim of violence
“We found a strong graded relationship between the breadth of exposure to abuse or household dysfunction during childhood and multiple risk factors for several of the leading causes of death in adults.” ~ The ACE Study
14
Felitti et al., 1998

## Slide 15
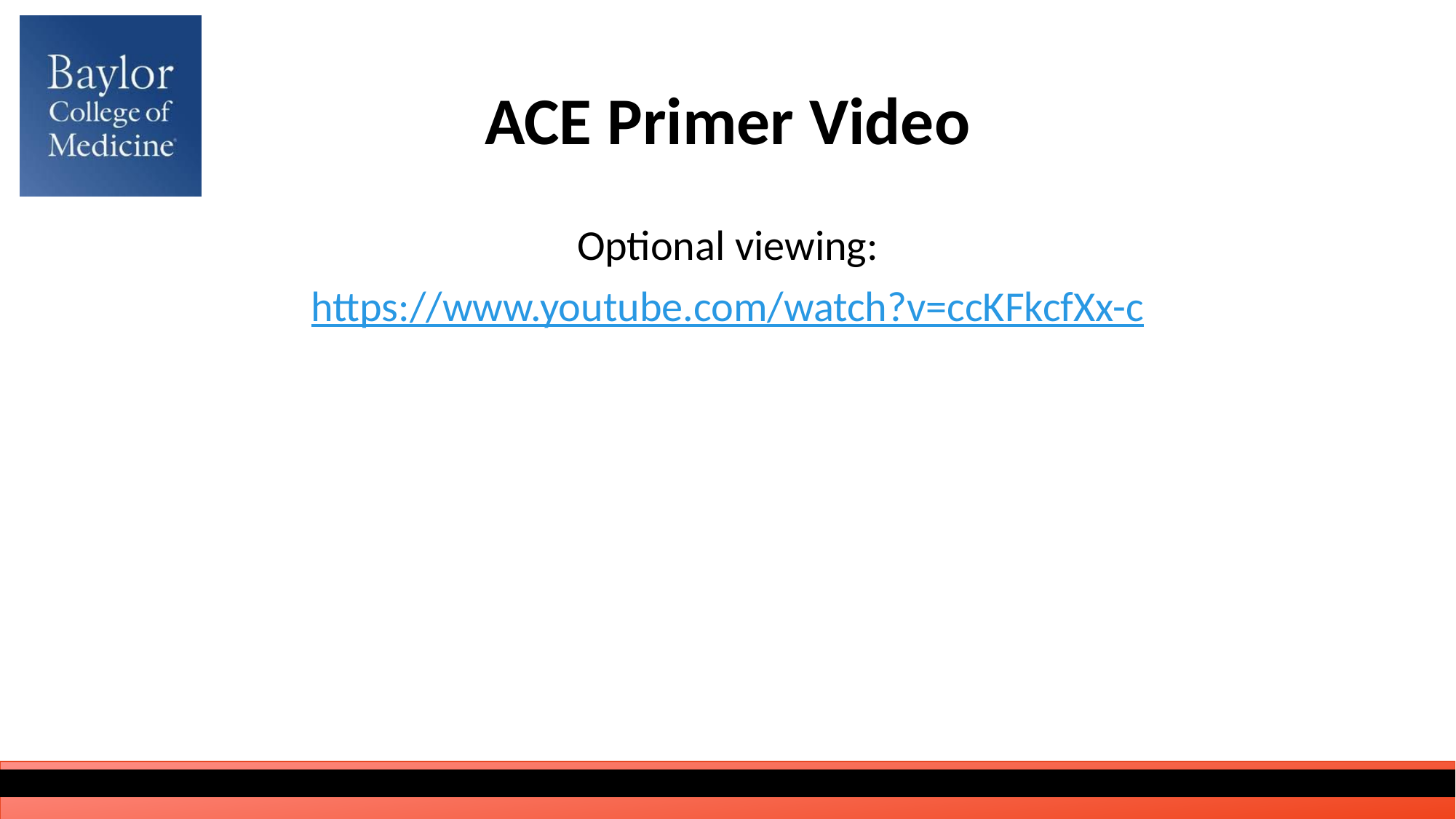

# ACE Primer Video
Optional viewing:
https://www.youtube.com/watch?v=ccKFkcfXx-c
15

## Slide 16
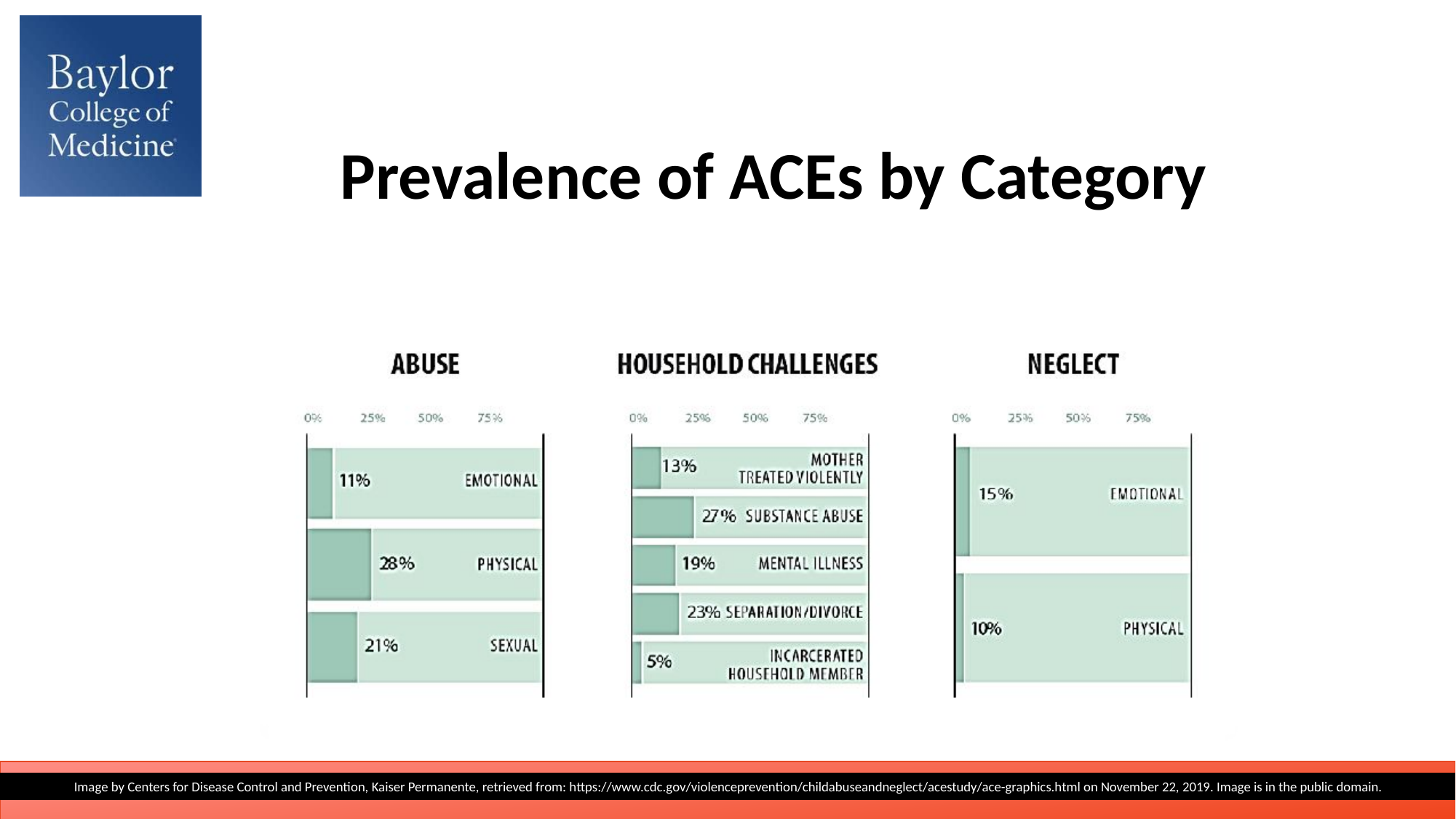

Prevalence of ACEs by Category
16
Image by Centers for Disease Control and Prevention, Kaiser Permanente, retrieved from: https://www.cdc.gov/violenceprevention/childabuseandneglect/acestudy/ace-graphics.html on November 22, 2019. Image is in the public domain.

## Slide 17
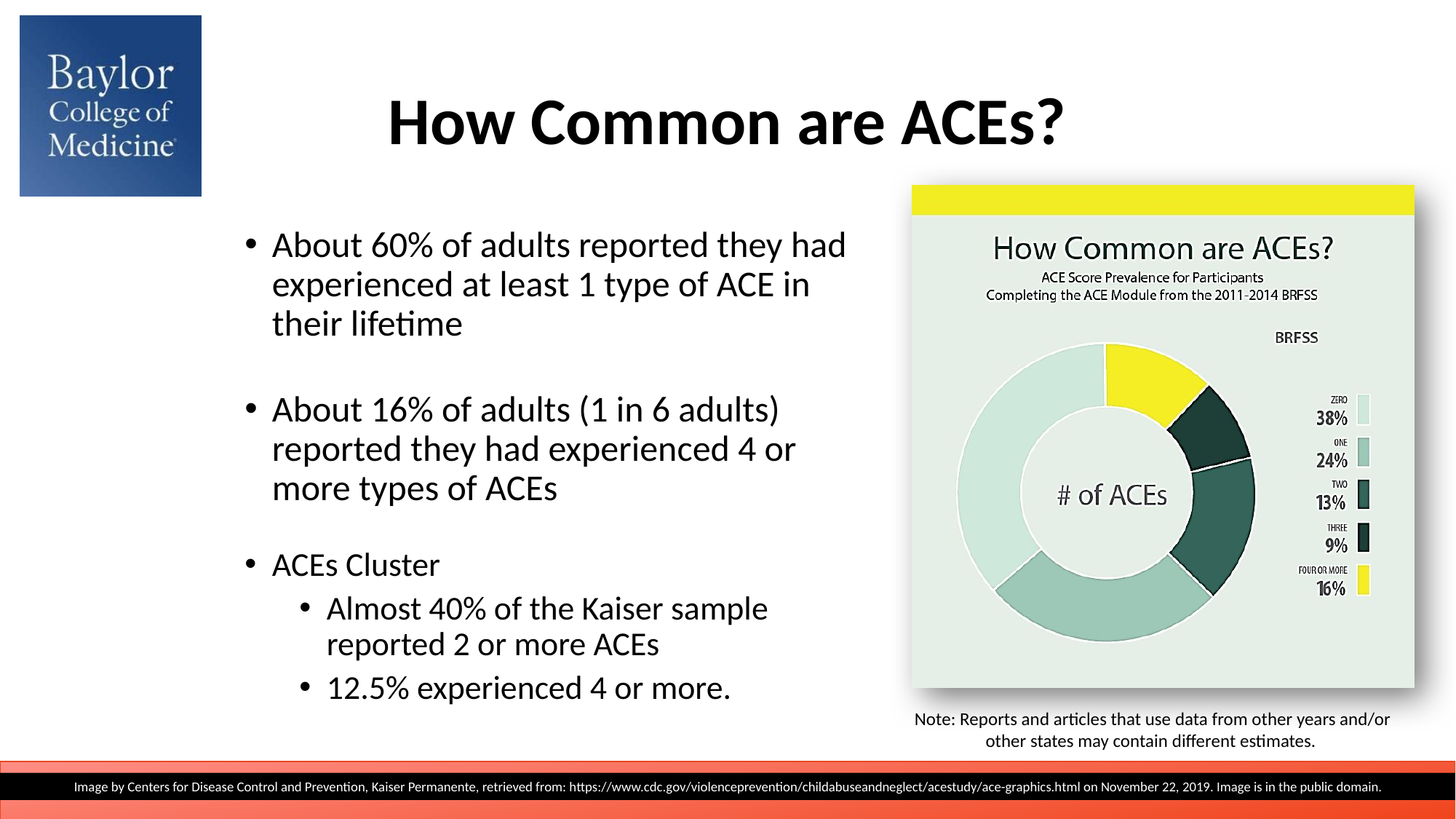

# How Common are ACEs?
About 60% of adults reported they had experienced at least 1 type of ACE in their lifetime
About 16% of adults (1 in 6 adults) reported they had experienced 4 or more types of ACEs
ACEs Cluster
Almost 40% of the Kaiser sample reported 2 or more ACEs
12.5% experienced 4 or more.
Note: Reports and articles that use data from other years and/or other states may contain different estimates.
17
Image by Centers for Disease Control and Prevention, Kaiser Permanente, retrieved from: https://www.cdc.gov/violenceprevention/childabuseandneglect/acestudy/ace-graphics.html on November 22, 2019. Image is in the public domain.

## Slide 18
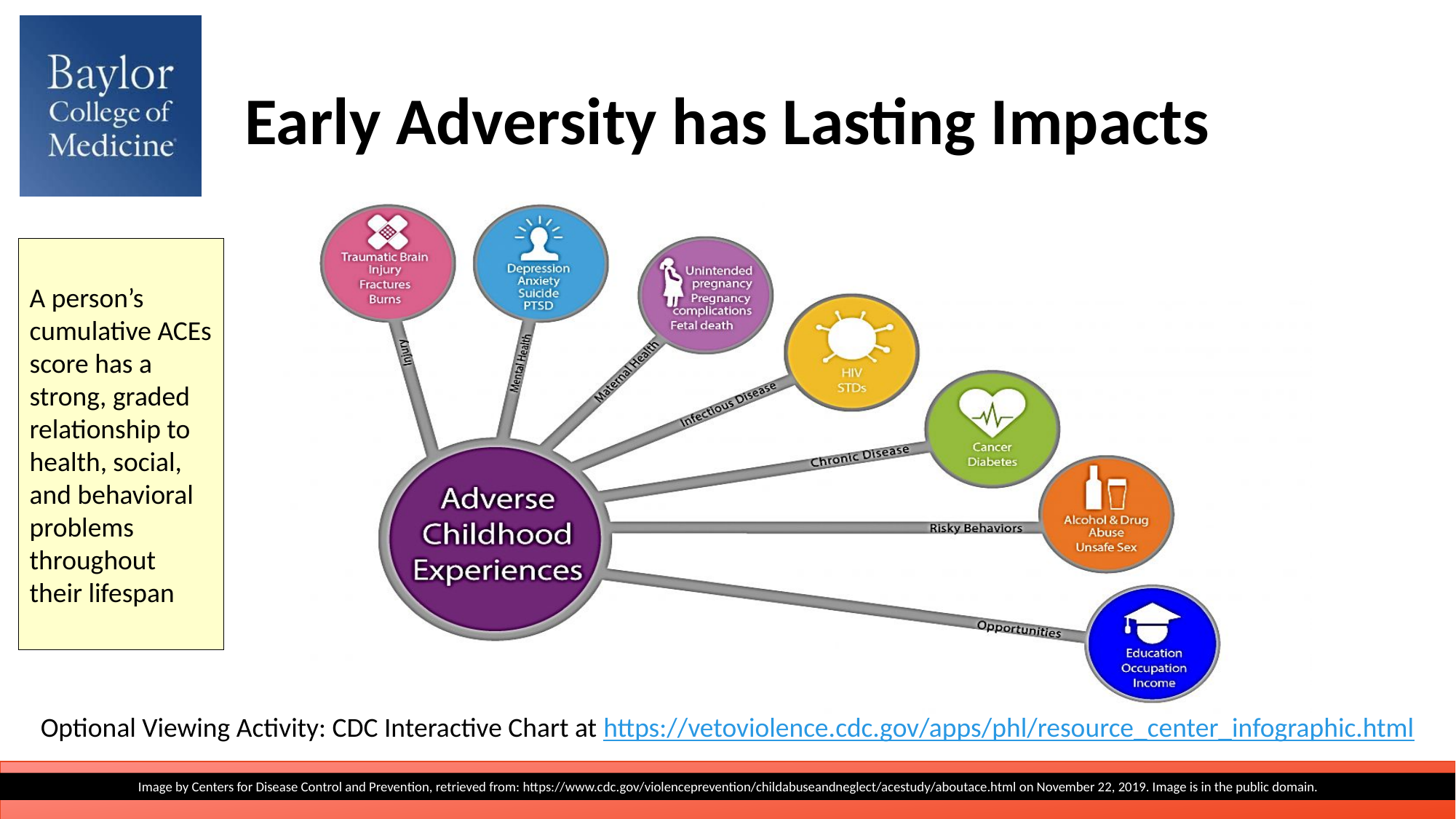

# Early Adversity has Lasting Impacts
A person’s cumulative ACEs score has a strong, graded relationship to health, social, and behavioral problems throughout their lifespan
Optional Viewing Activity: CDC Interactive Chart at https://vetoviolence.cdc.gov/apps/phl/resource_center_infographic.html
18
Image by Centers for Disease Control and Prevention, retrieved from: https://www.cdc.gov/violenceprevention/childabuseandneglect/acestudy/aboutace.html on November 22, 2019. Image is in the public domain.

## Slide 19
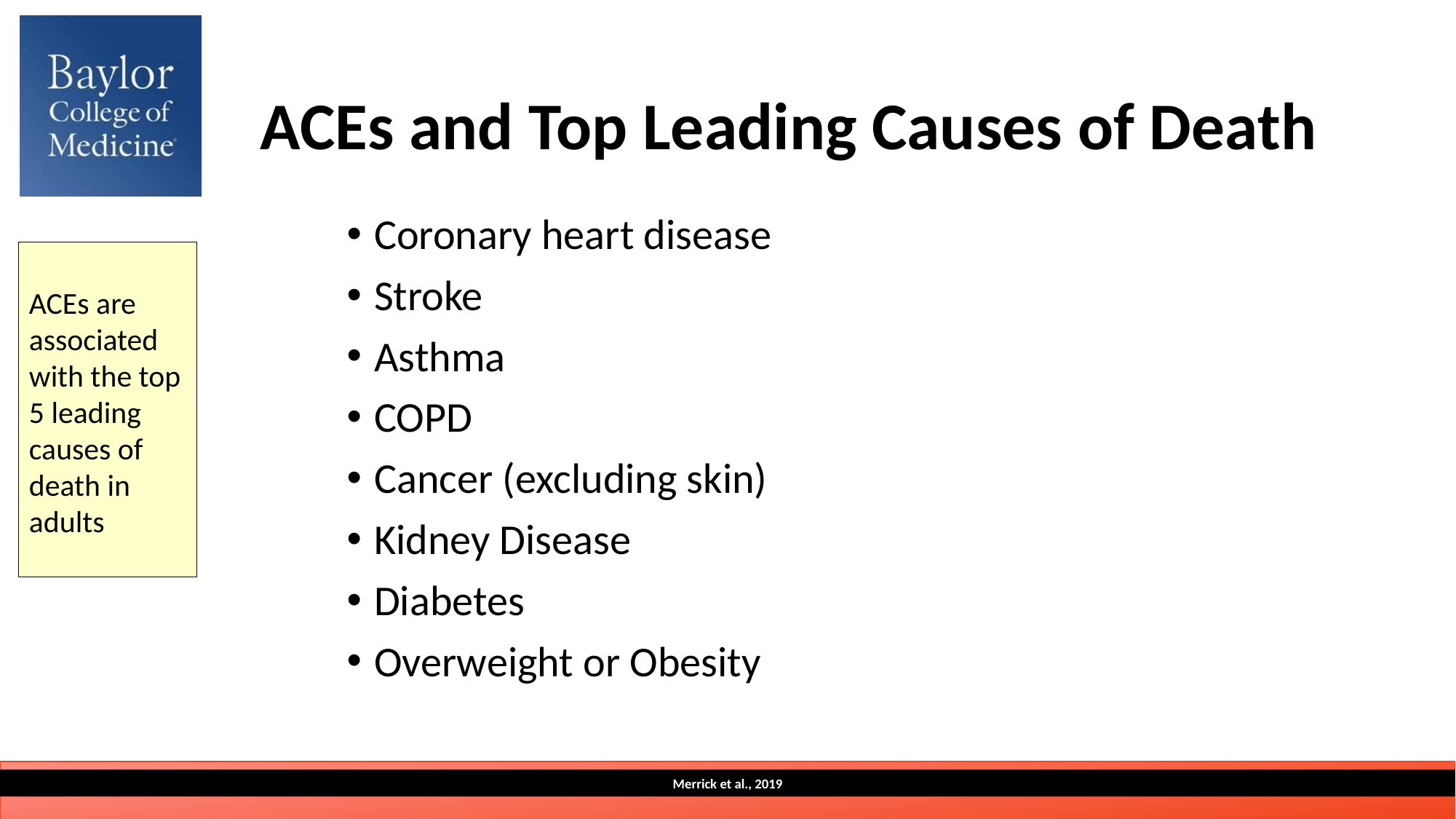

# ACEs and Top Leading Causes of Death
Coronary heart disease
Stroke
Asthma
COPD
Cancer (excluding skin)
Kidney Disease
Diabetes
Overweight or Obesity
ACEs are associated with the top 5 leading causes of death in adults
19
Merrick et al., 2019

## Slide 20
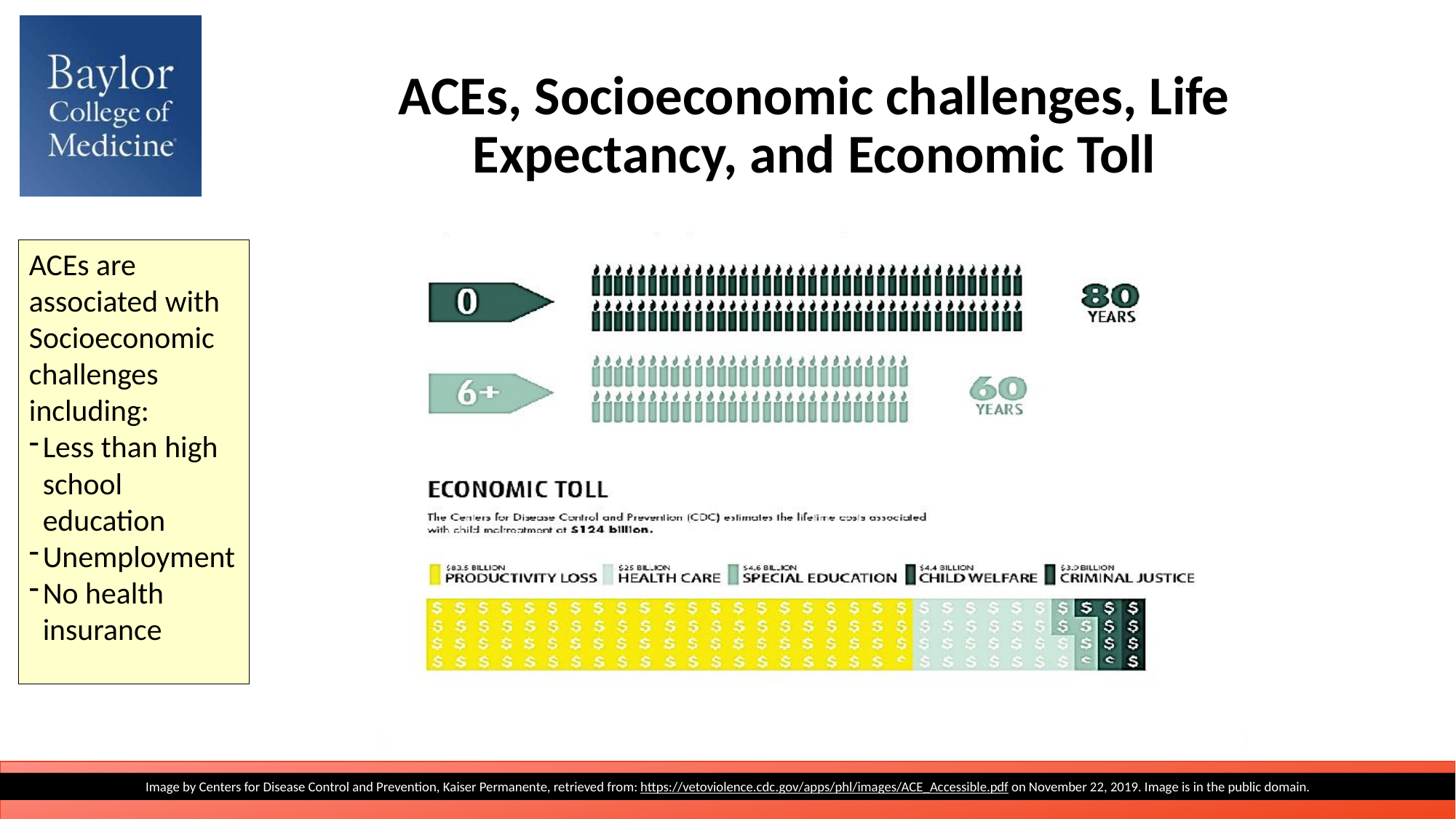

# ACEs, Socioeconomic challenges, Life Expectancy, and Economic Toll
ACEs are associated with Socioeconomic challenges including:
Less than high school education
Unemployment
No health insurance
20
Image by Centers for Disease Control and Prevention, Kaiser Permanente, retrieved from: https://vetoviolence.cdc.gov/apps/phl/images/ACE_Accessible.pdf on November 22, 2019. Image is in the public domain.

## Slide 21
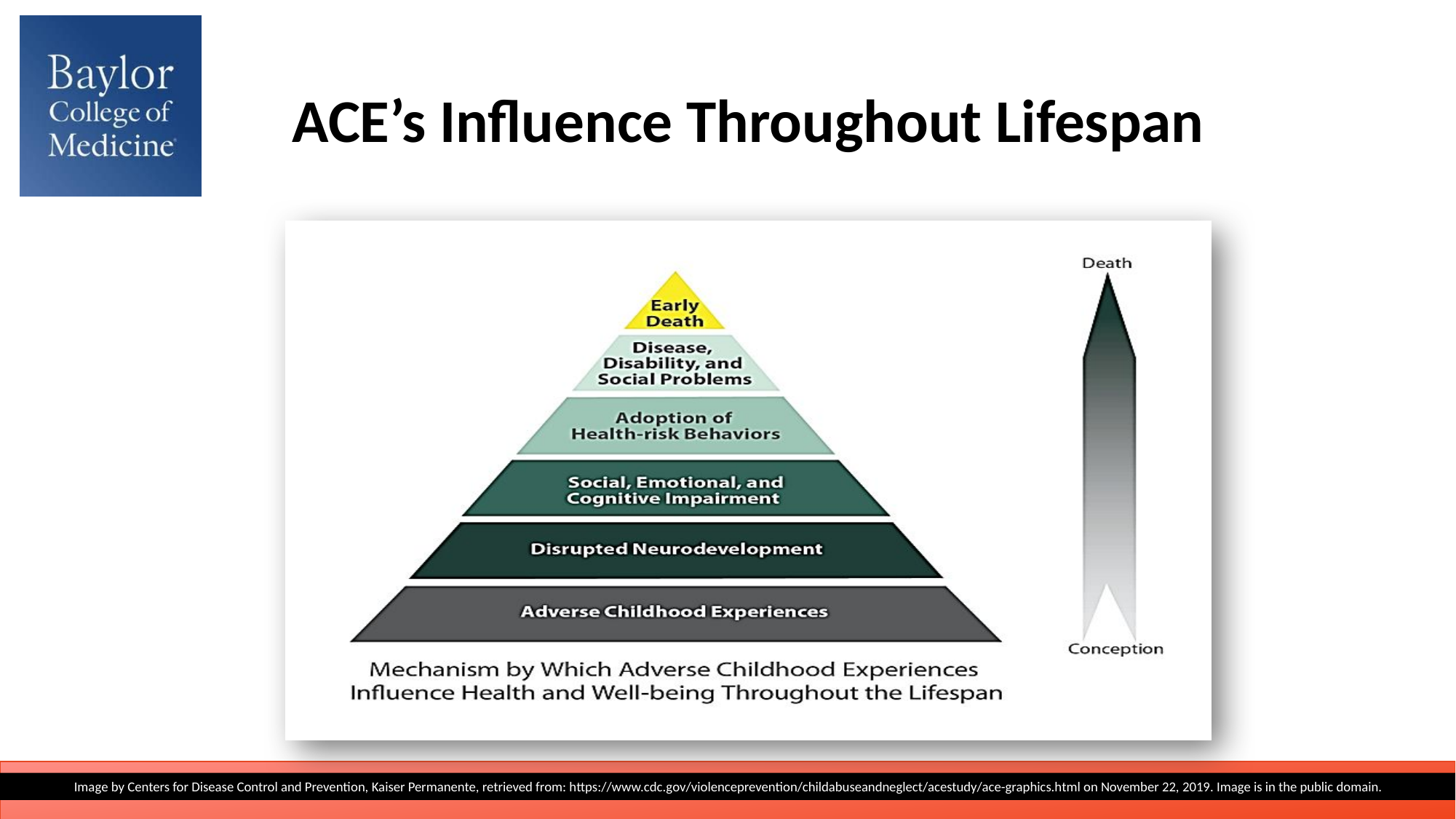

# ACE’s Influence Throughout Lifespan
21
Image by Centers for Disease Control and Prevention, Kaiser Permanente, retrieved from: https://www.cdc.gov/violenceprevention/childabuseandneglect/acestudy/ace-graphics.html on November 22, 2019. Image is in the public domain.

## Slide 22
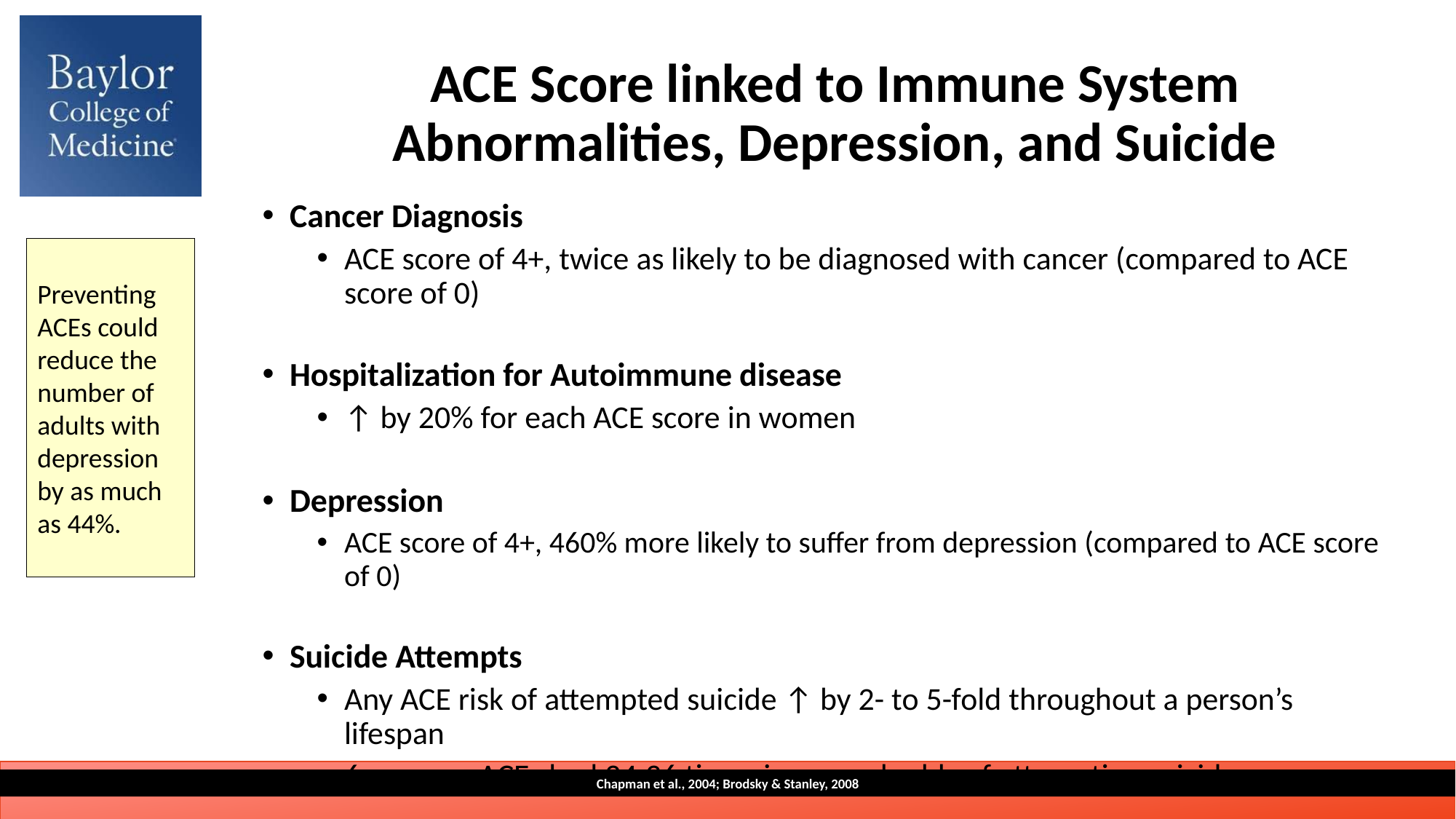

# ACE Score linked to Immune System Abnormalities, Depression, and Suicide
Cancer Diagnosis
ACE score of 4+, twice as likely to be diagnosed with cancer (compared to ACE score of 0)
Hospitalization for Autoimmune disease
↑ by 20% for each ACE score in women
Depression
ACE score of 4+, 460% more likely to suffer from depression (compared to ACE score of 0)
Suicide Attempts
Any ACE risk of attempted suicide ↑ by 2- to 5-fold throughout a person’s lifespan
6 or more ACEs had 24.36 times increased odds of attempting suicide
Preventing ACEs could reduce the number of adults with depression by as much as 44%.
22
Chapman et al., 2004; Brodsky & Stanley, 2008

## Slide 23
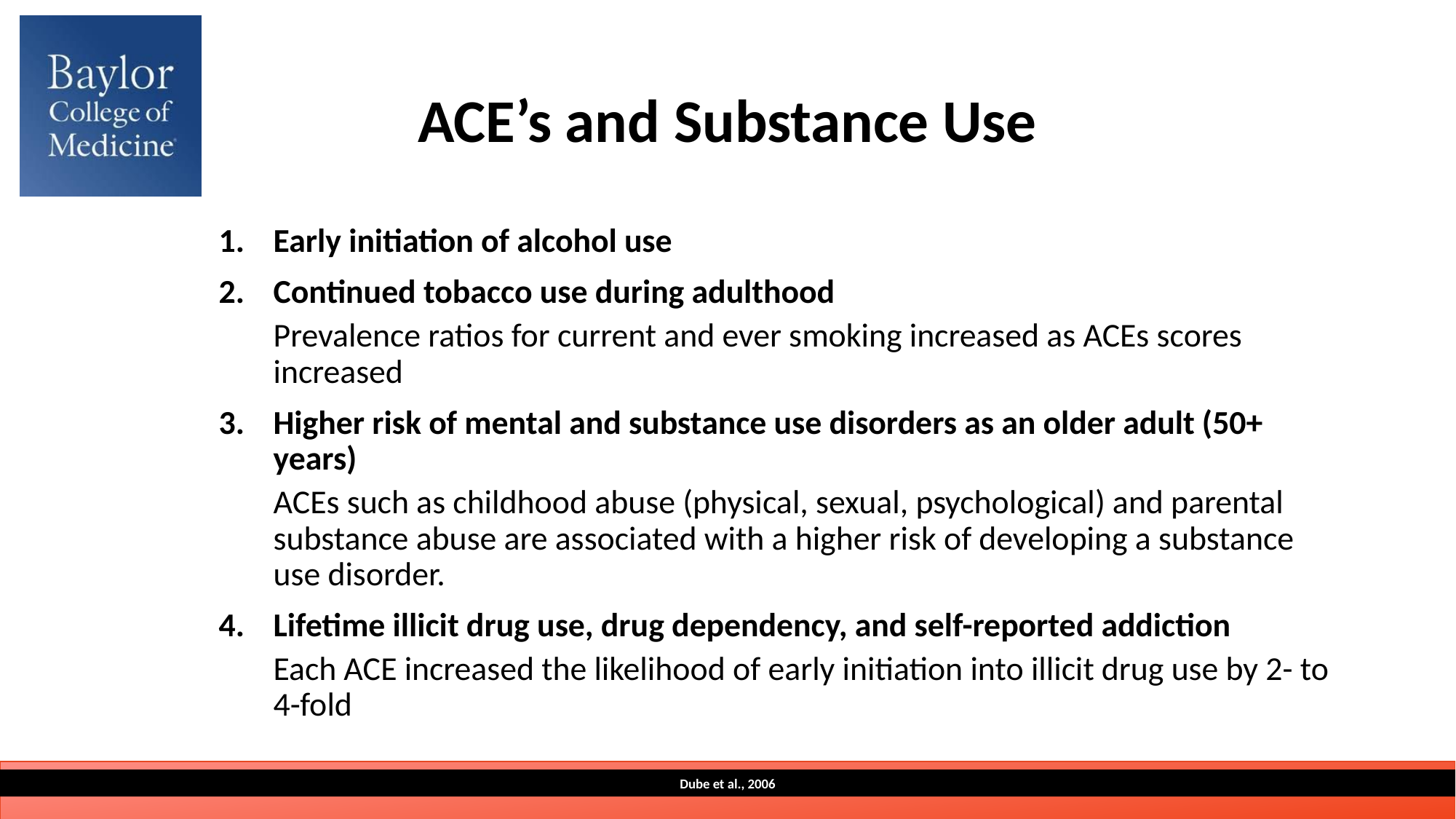

# ACE’s and Substance Use
Early initiation of alcohol use
Continued tobacco use during adulthood
Prevalence ratios for current and ever smoking increased as ACEs scores increased
Higher risk of mental and substance use disorders as an older adult (50+ years)
ACEs such as childhood abuse (physical, sexual, psychological) and parental substance abuse are associated with a higher risk of developing a substance use disorder.
Lifetime illicit drug use, drug dependency, and self-reported addiction
Each ACE increased the likelihood of early initiation into illicit drug use by 2- to 4-fold
23
Dube et al., 2006

## Slide 24
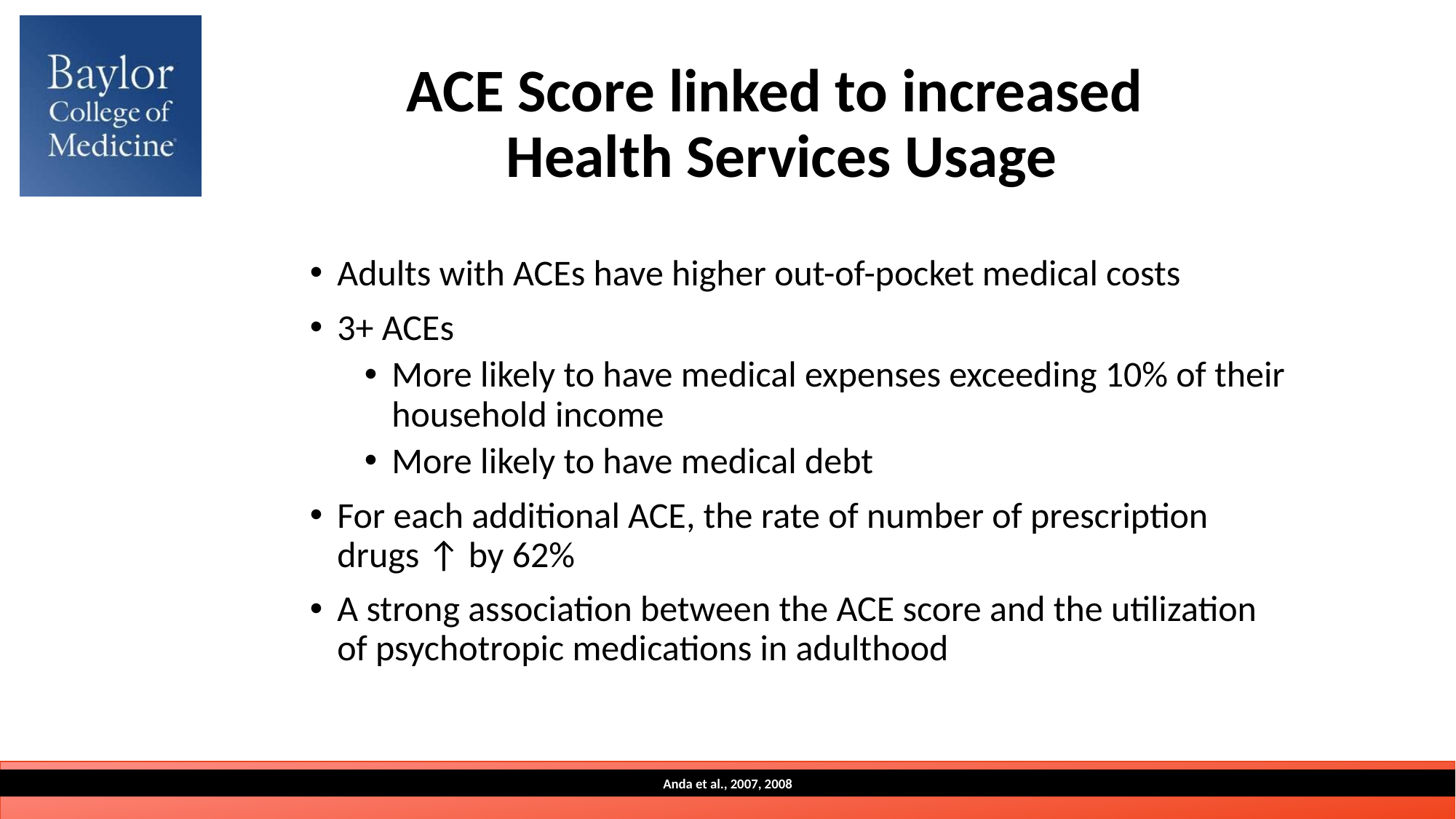

# ACE Score linked to increased Health Services Usage
Adults with ACEs have higher out-of-pocket medical costs
3+ ACEs
More likely to have medical expenses exceeding 10% of their household income
More likely to have medical debt
For each additional ACE, the rate of number of prescription drugs ↑ by 62%
A strong association between the ACE score and the utilization of psychotropic medications in adulthood
24
Anda et al., 2007, 2008

## Slide 25
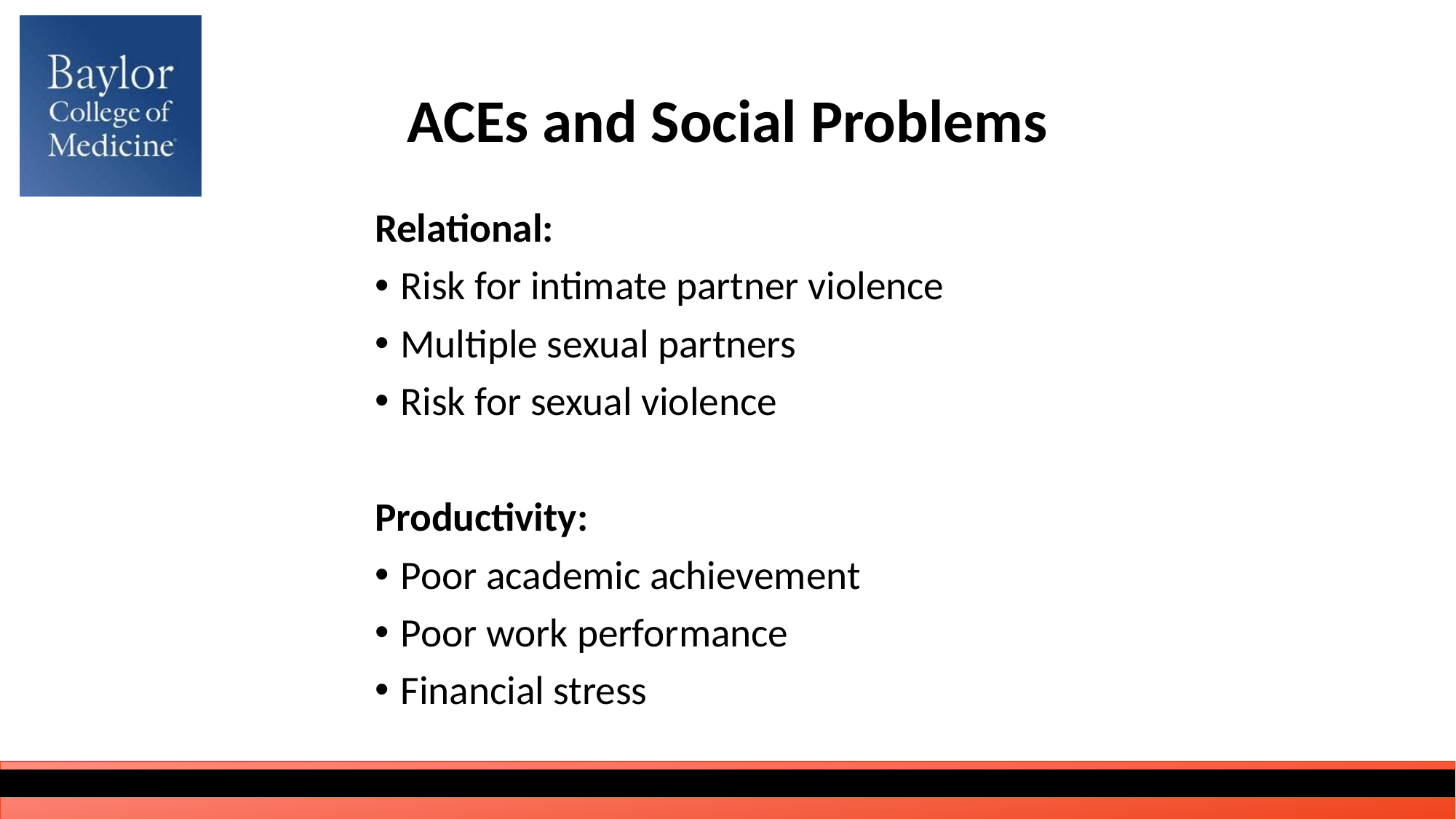

# ACEs and Social Problems
Relational:
Risk for intimate partner violence
Multiple sexual partners
Risk for sexual violence
Productivity:
Poor academic achievement
Poor work performance
Financial stress
25

## Slide 26
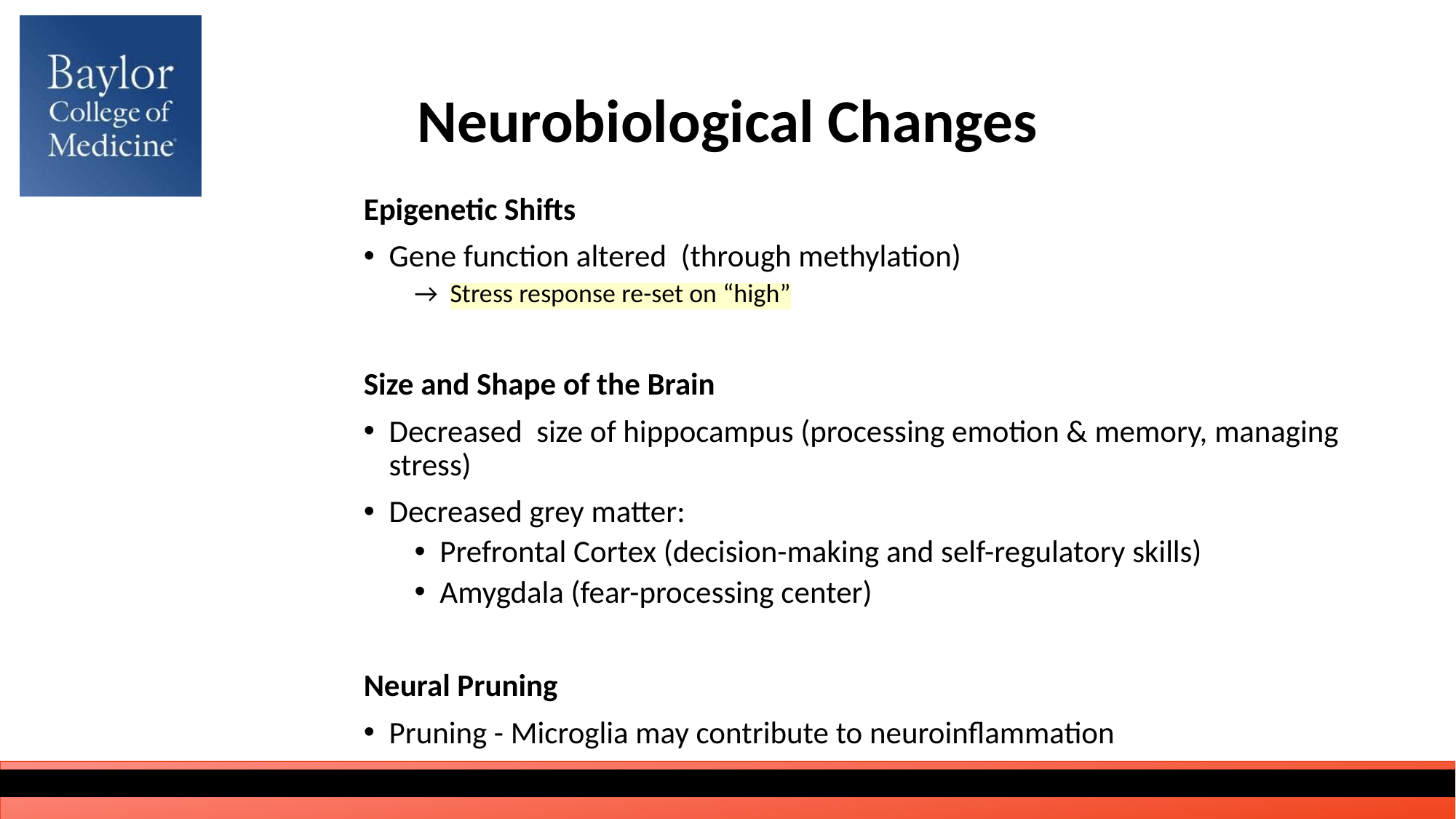

# Neurobiological Changes
Epigenetic Shifts
Gene function altered (through methylation)
→ Stress response re-set on “high”
Size and Shape of the Brain
Decreased size of hippocampus (processing emotion & memory, managing stress)
Decreased grey matter:
Prefrontal Cortex (decision-making and self-regulatory skills)
Amygdala (fear-processing center)
Neural Pruning
Pruning - Microglia may contribute to neuroinflammation
26

## Slide 27
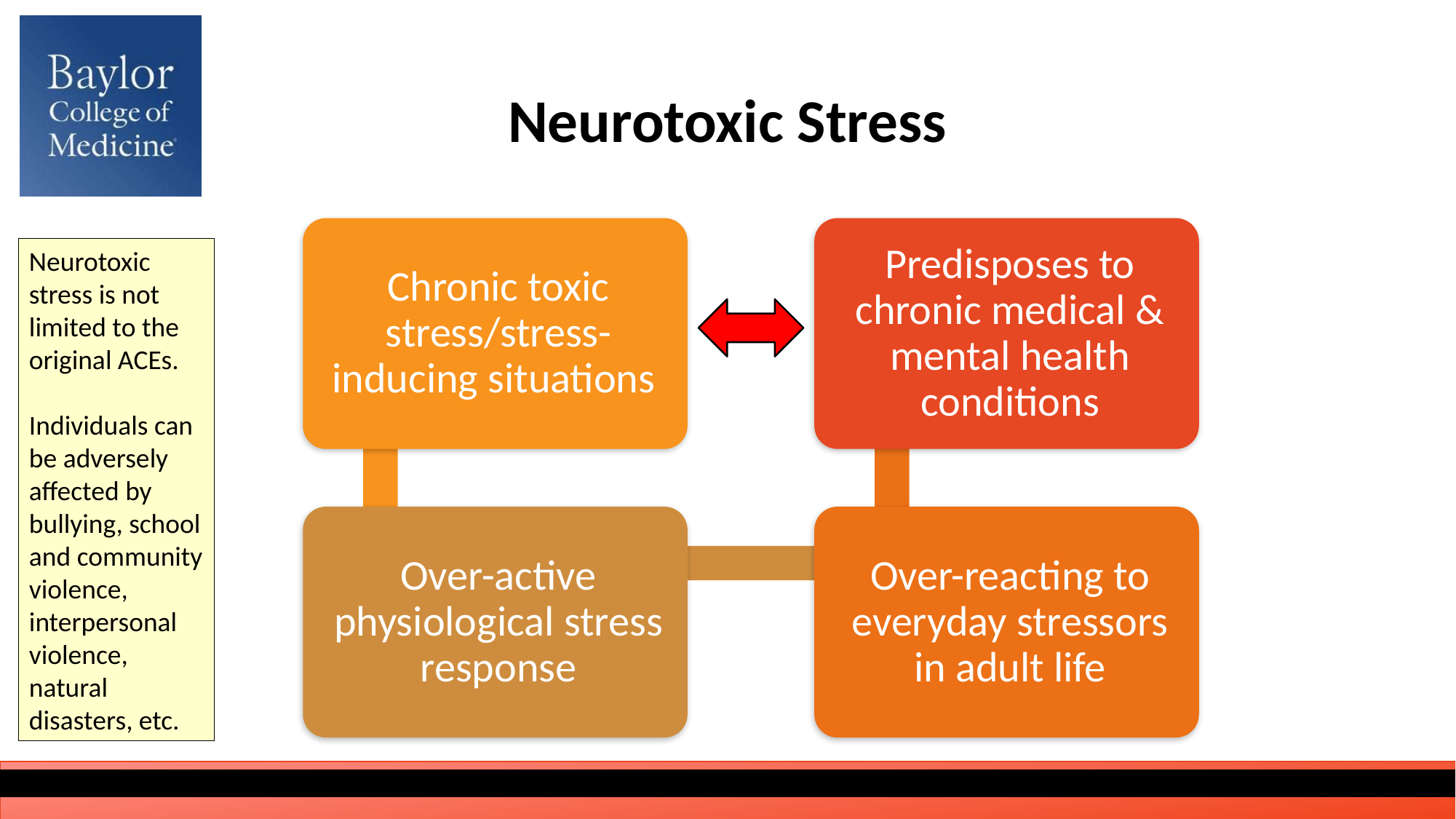

# Neurotoxic Stress
Neurotoxic stress is not limited to the original ACEs.
Individuals can be adversely affected by bullying, school and community violence, interpersonal violence, natural disasters, etc.
27

## Slide 28
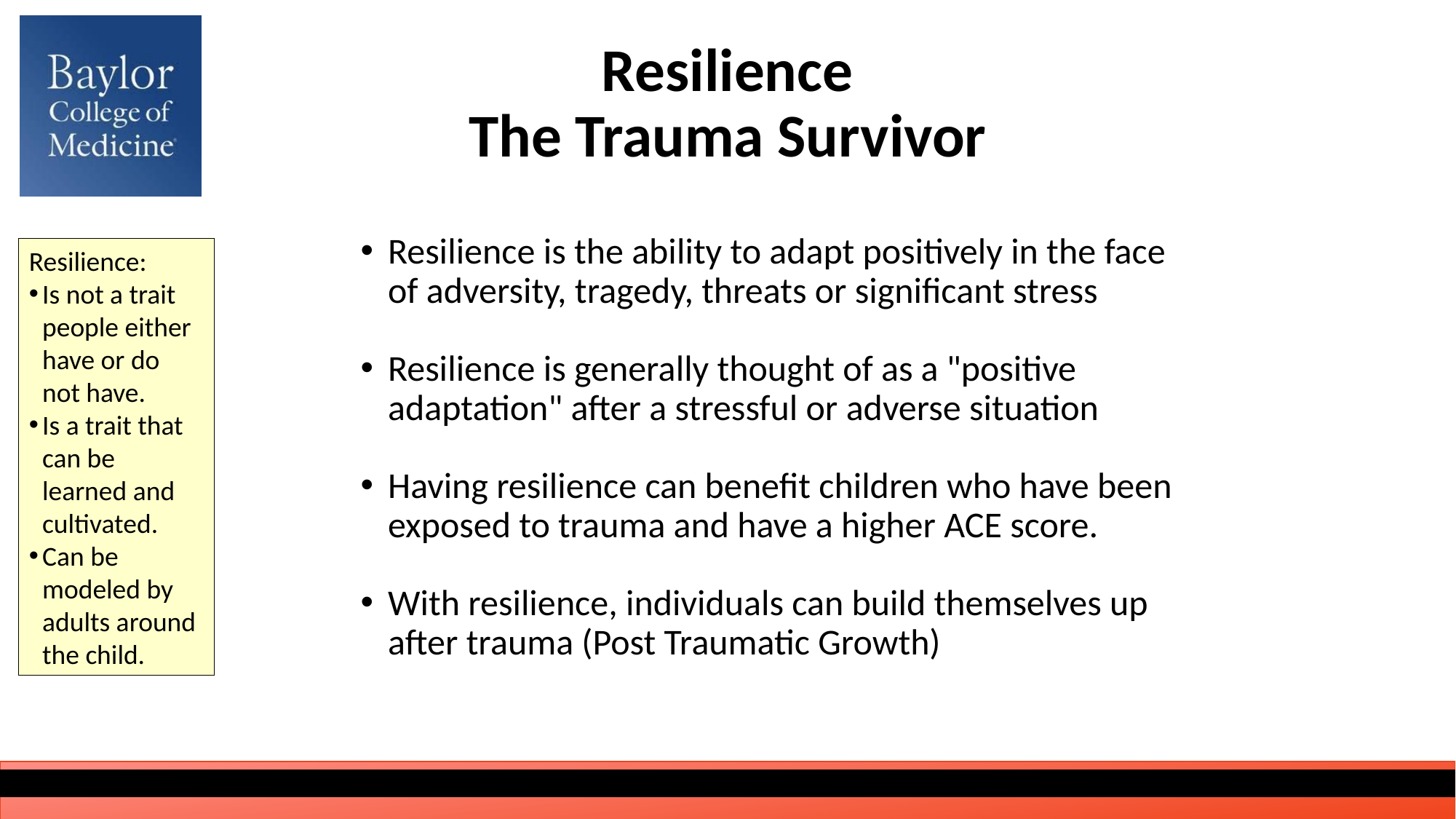

# Resilience The Trauma Survivor
Resilience is the ability to adapt positively in the face of adversity, tragedy, threats or significant stress
Resilience is generally thought of as a "positive adaptation" after a stressful or adverse situation
Having resilience can benefit children who have been exposed to trauma and have a higher ACE score.
With resilience, individuals can build themselves up after trauma (Post Traumatic Growth)
Resilience:
Is not a trait people either have or do not have.
Is a trait that can be learned and cultivated.
Can be modeled by adults around the child.
28

## Slide 29
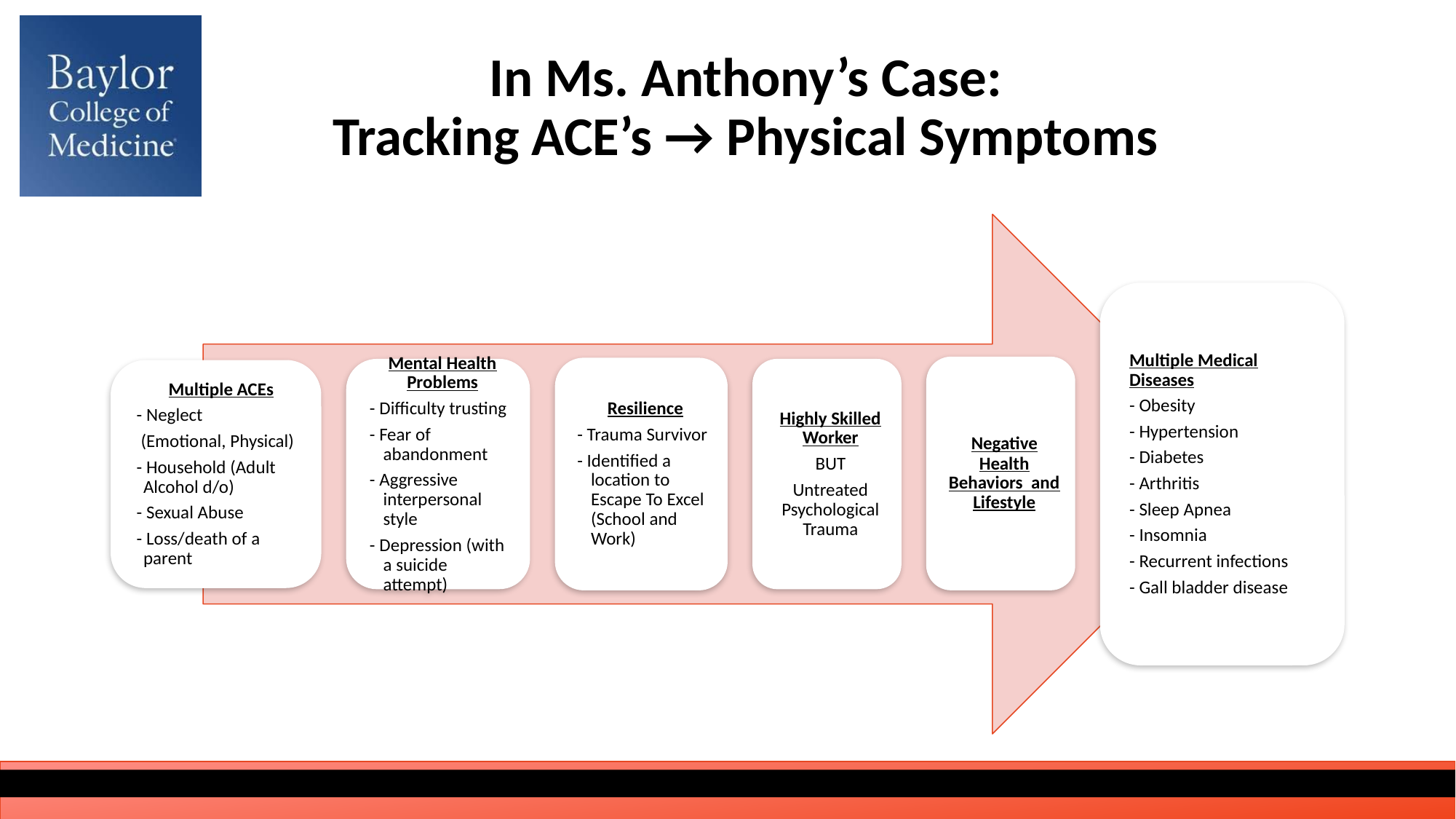

# In Ms. Anthony’s Case: Tracking ACE’s → Physical Symptoms
29

## Slide 30
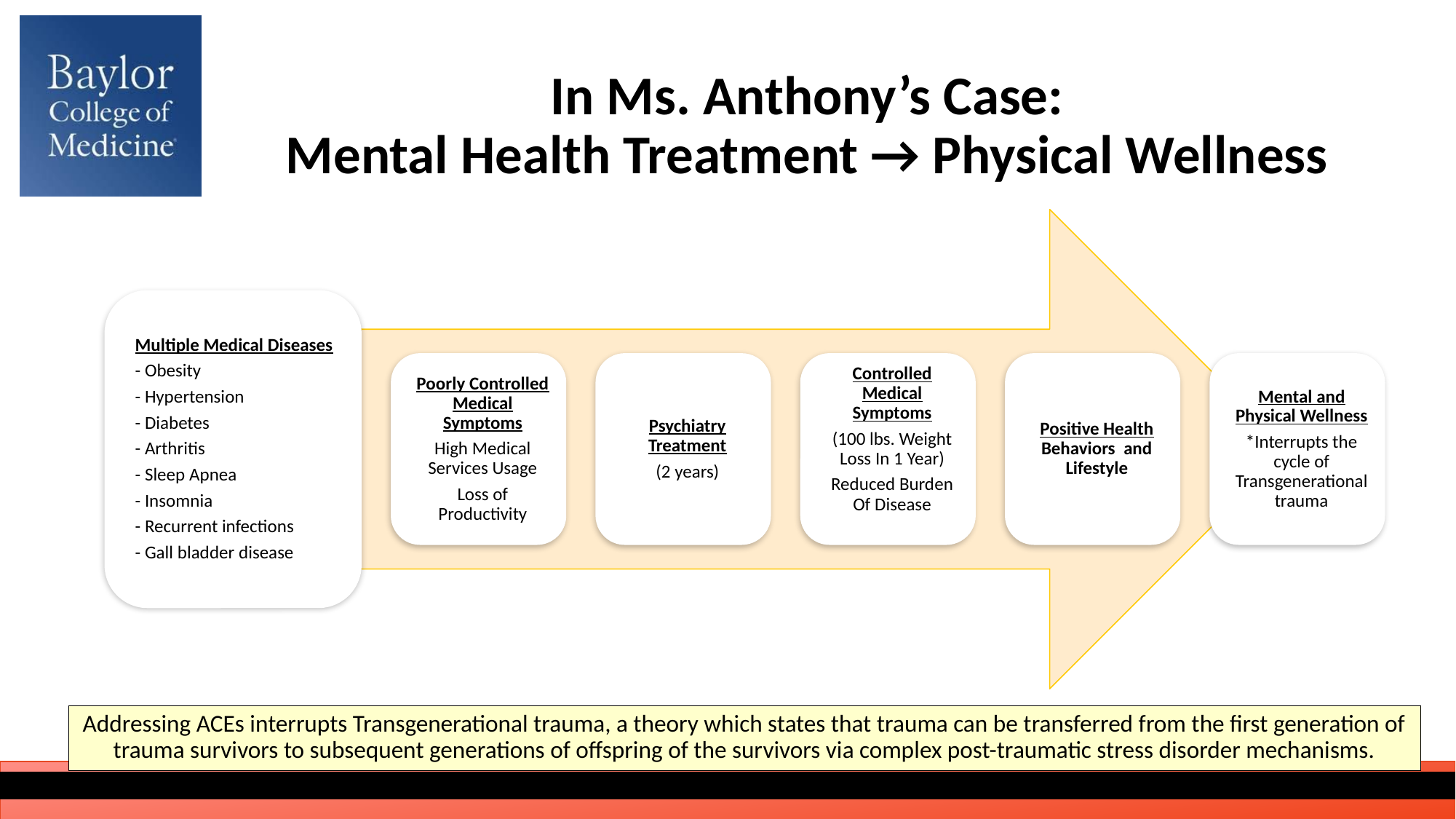

In Ms. Anthony’s Case:
Mental Health Treatment → Physical Wellness
Addressing ACEs interrupts Transgenerational trauma, a theory which states that trauma can be transferred from the first generation of trauma survivors to subsequent generations of offspring of the survivors via complex post-traumatic stress disorder mechanisms.
30

## Slide 31
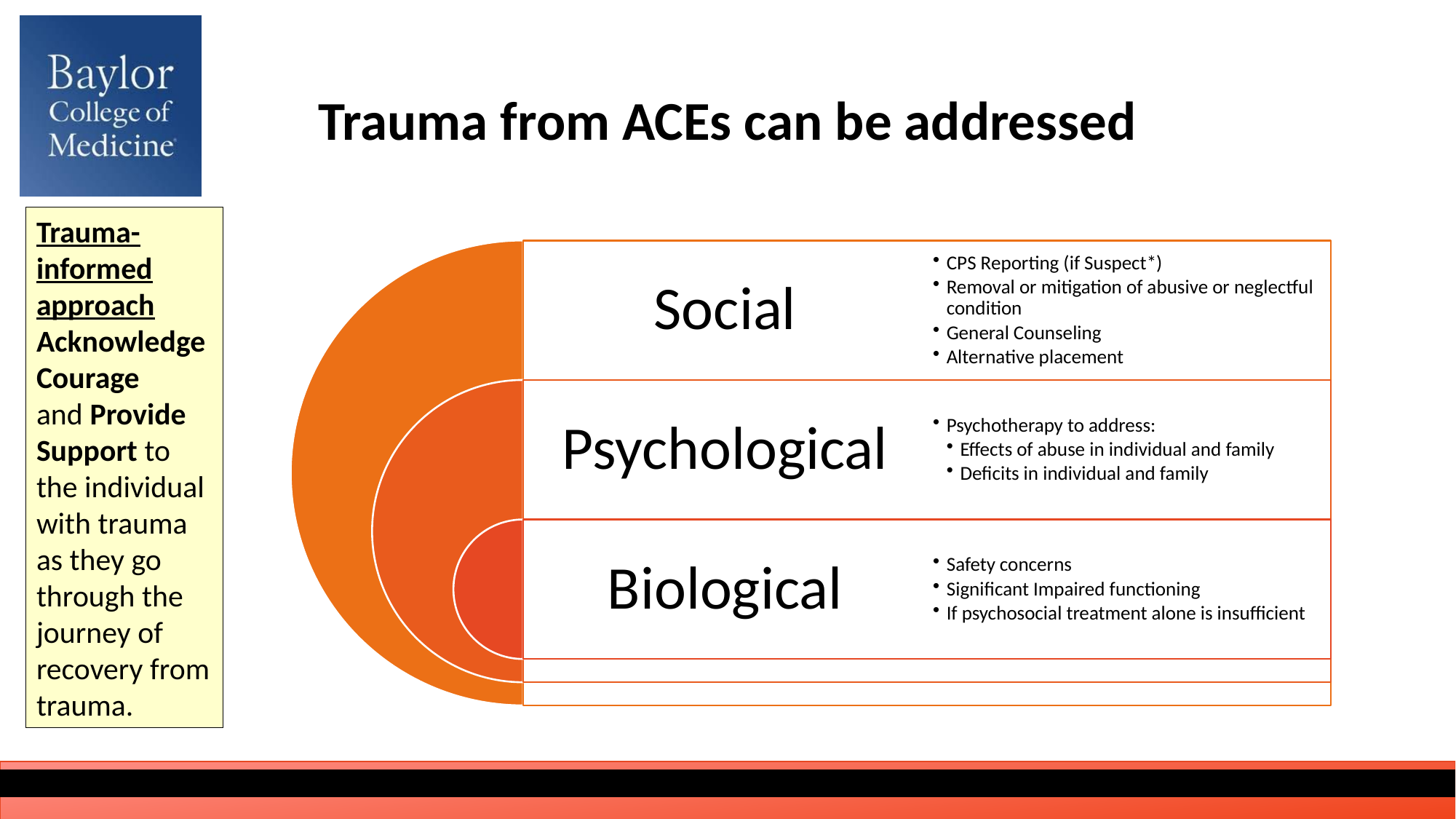

# Trauma from ACEs can be addressed
Trauma-informed approach
Acknowledge Courage
and Provide Support to the individual with trauma as they go through the journey of recovery from trauma.
31

## Slide 32
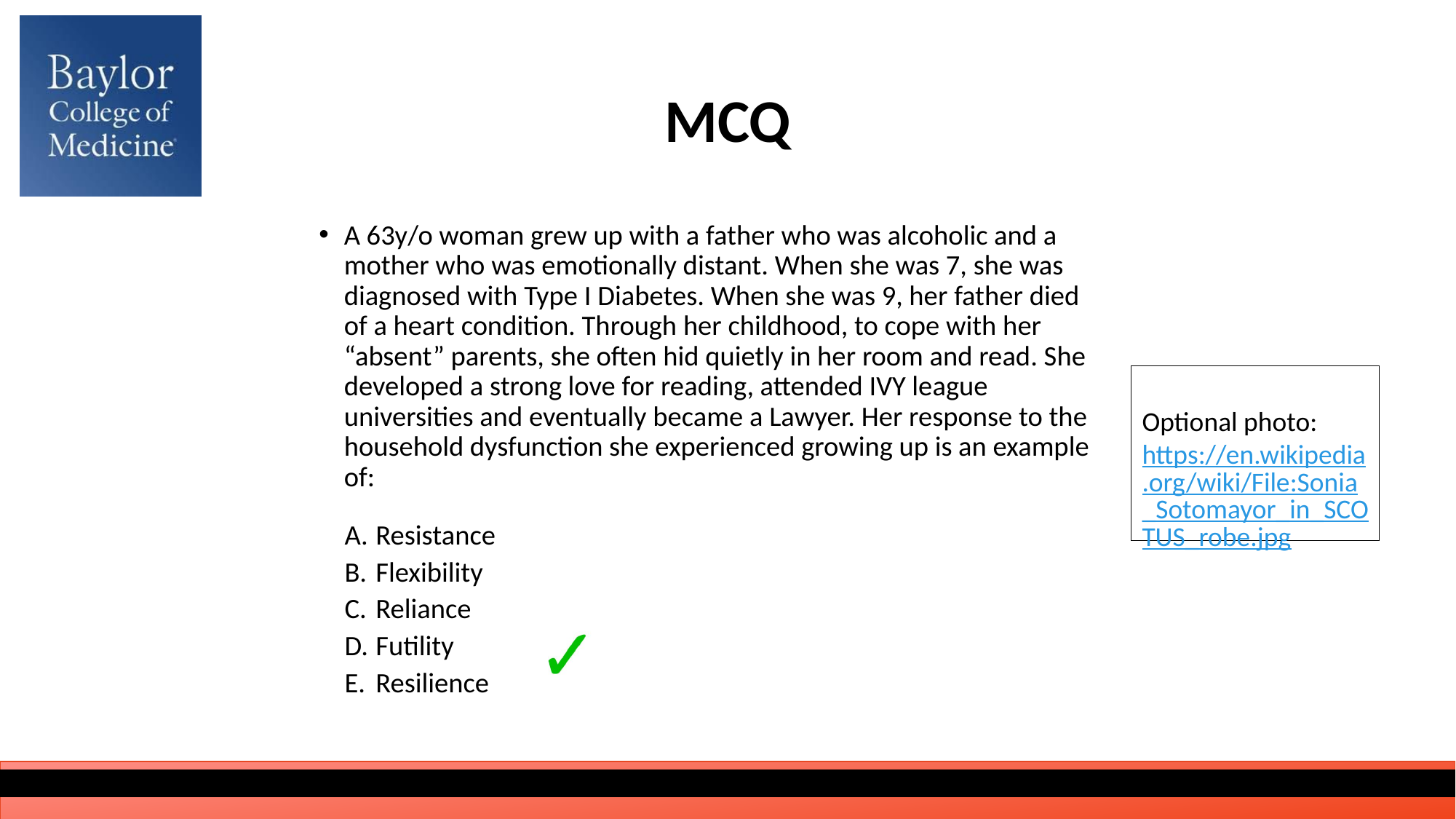

# MCQ
A 63y/o woman grew up with a father who was alcoholic and a mother who was emotionally distant. When she was 7, she was diagnosed with Type I Diabetes. When she was 9, her father died of a heart condition. Through her childhood, to cope with her “absent” parents, she often hid quietly in her room and read. She developed a strong love for reading, attended IVY league universities and eventually became a Lawyer. Her response to the household dysfunction she experienced growing up is an example of:
Resistance
Flexibility
Reliance
Futility
Resilience
Optional photo: https://en.wikipedia.org/wiki/File:Sonia_Sotomayor_in_SCOTUS_robe.jpg
32

## Slide 33
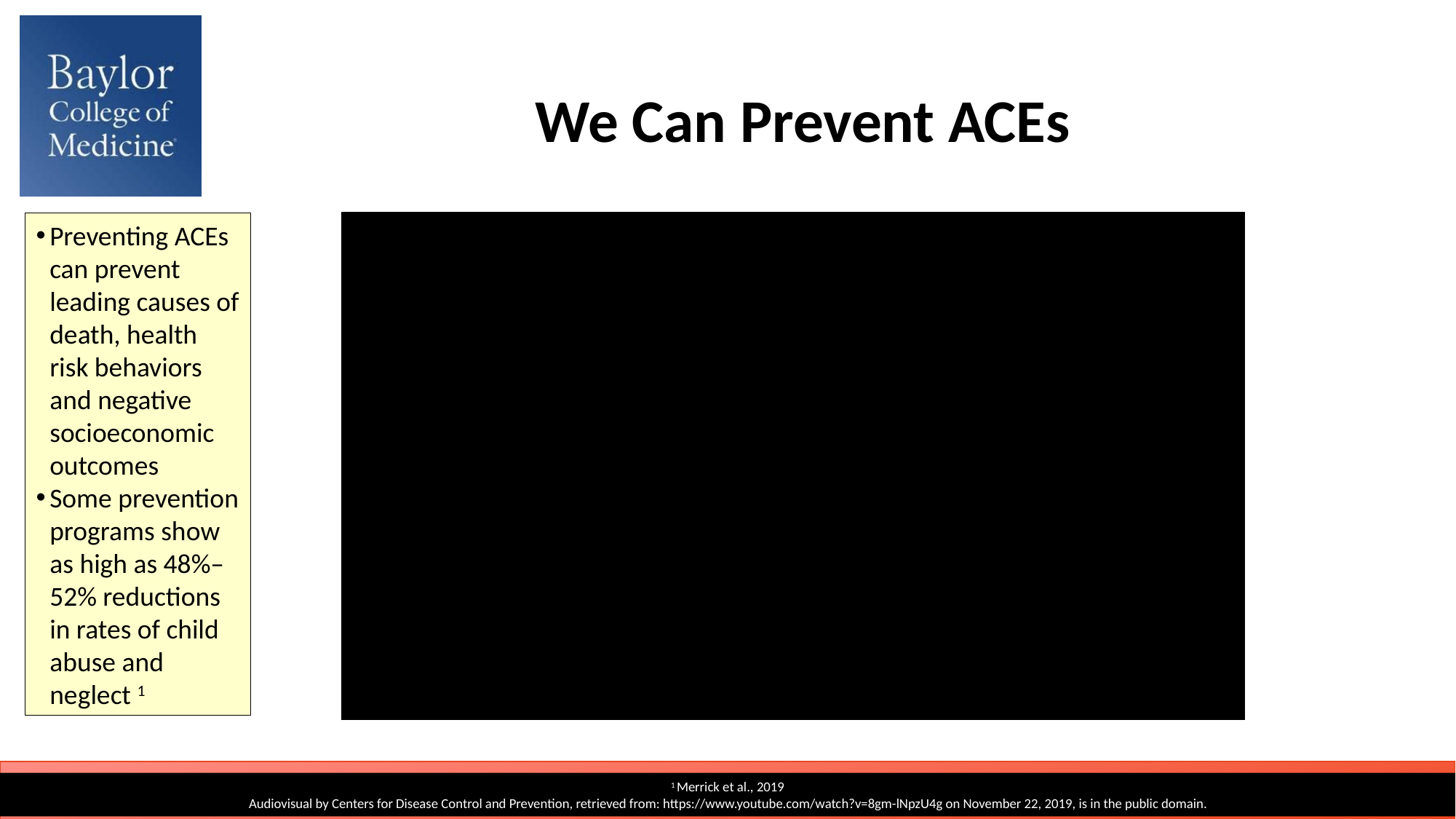

# We Can Prevent ACEs
Preventing ACEs can prevent leading causes of death, health risk behaviors and negative socioeconomic outcomes
Some prevention programs show as high as 48%–52% reductions in rates of child abuse and neglect 1
33
1 Merrick et al., 2019
Audiovisual by Centers for Disease Control and Prevention, retrieved from: https://www.youtube.com/watch?v=8gm-lNpzU4g on November 22, 2019, is in the public domain.

## Slide 34
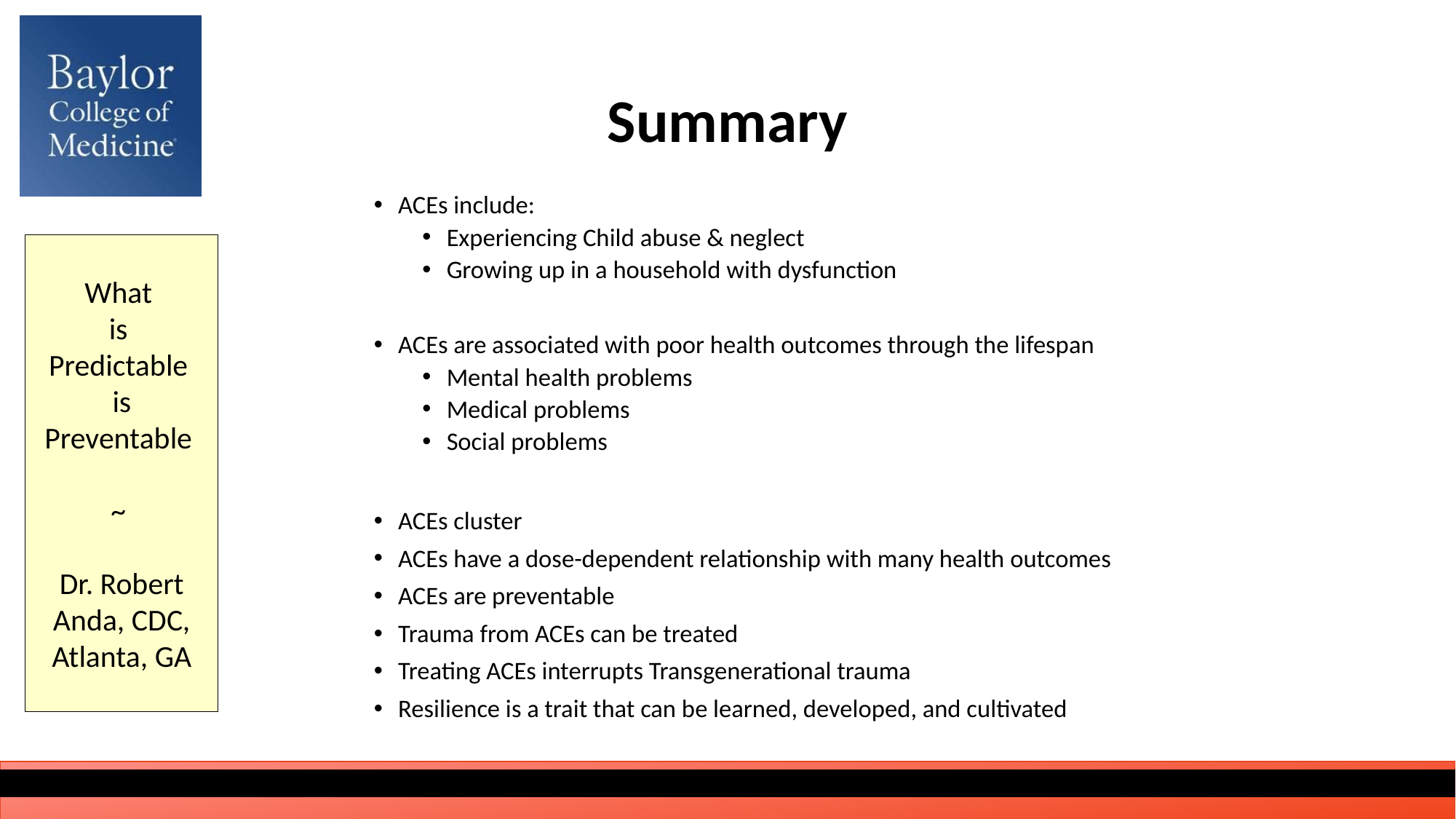

# Summary
ACEs include:
Experiencing Child abuse & neglect
Growing up in a household with dysfunction
ACEs are associated with poor health outcomes through the lifespan
Mental health problems
Medical problems
Social problems
ACEs cluster
ACEs have a dose-dependent relationship with many health outcomes
ACEs are preventable
Trauma from ACEs can be treated
Treating ACEs interrupts Transgenerational trauma
Resilience is a trait that can be learned, developed, and cultivated
What
is
Predictable
is Preventable
~
Dr. Robert Anda, CDC, Atlanta, GA
34

## Slide 35
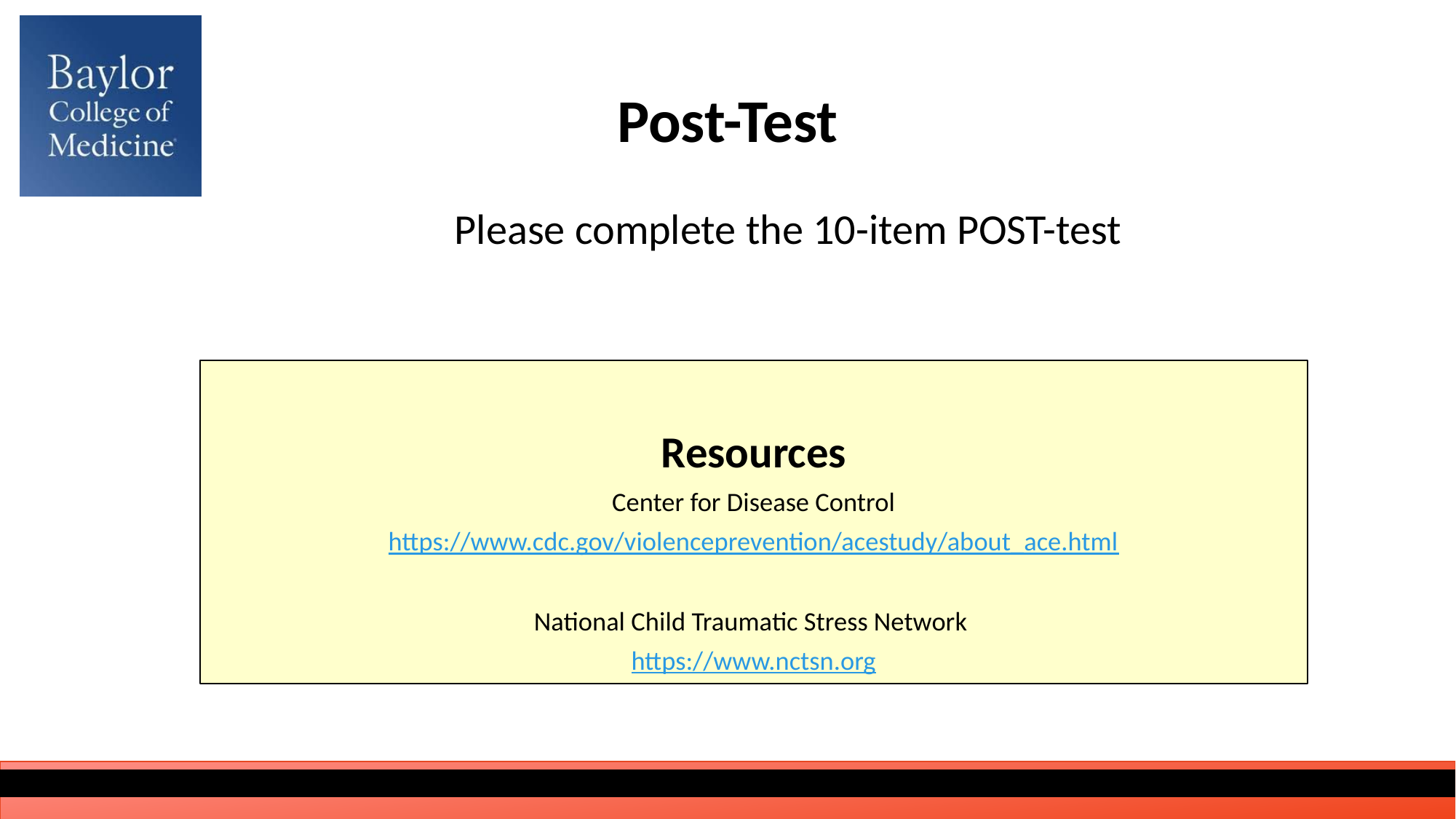

# Post-Test
Please complete the 10-item POST-test
Resources
Center for Disease Control
https://www.cdc.gov/violenceprevention/acestudy/about_ace.html
National Child Traumatic Stress Network
https://www.nctsn.org
35

## Slide 36
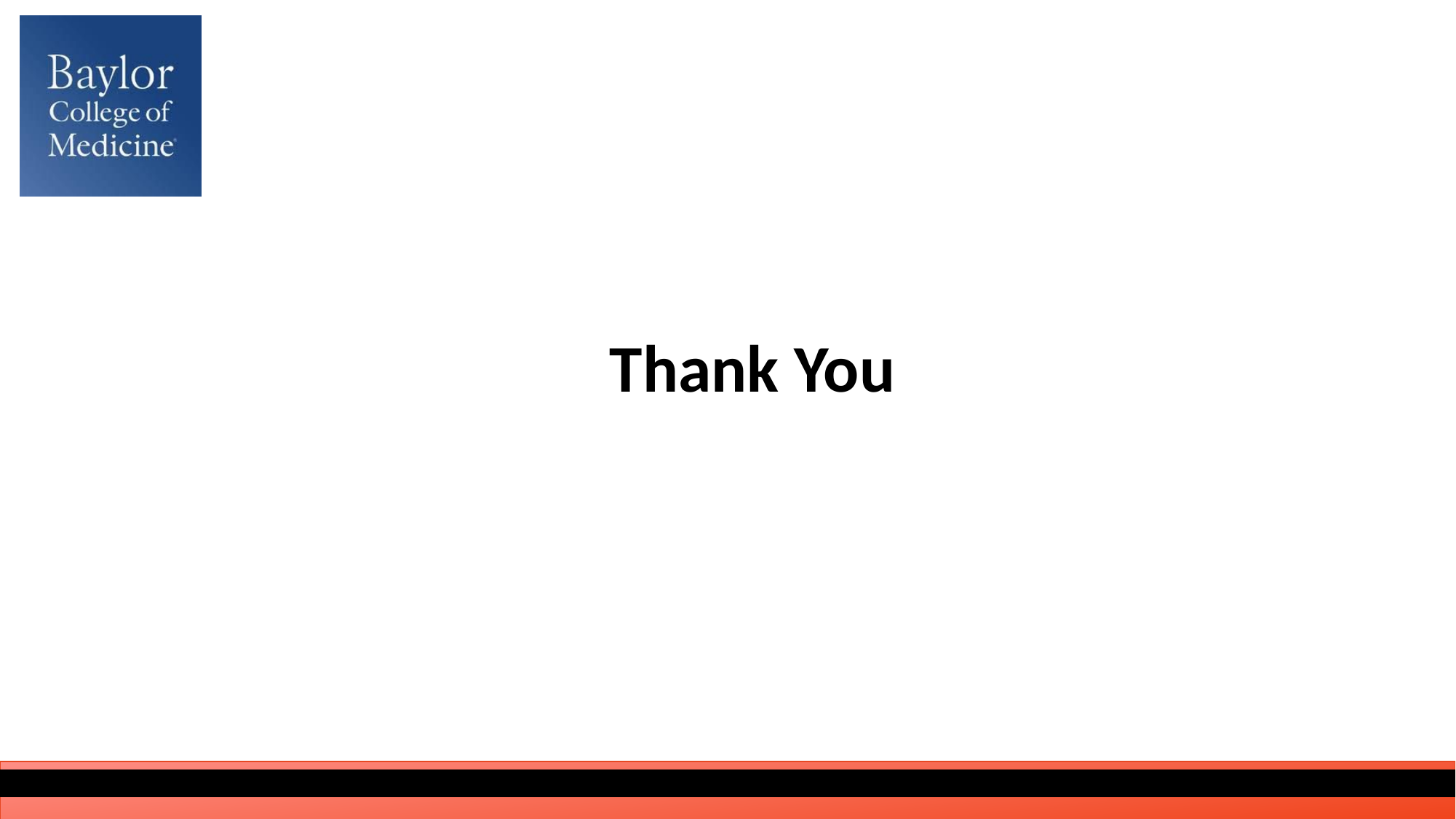

# Thank You
36
