# Supplementary material for: Introducing ACEs (Adverse Childhood Experiences) and Resilience to First-Year Medical Students
Source: MedEdPORTAL. 2020 Sep 15;16:10964. doi: 10.15766/mep_2374-8265.10964 (PMC7499813; doi:10.15766/mep_2374-8265.10964)
Supplement: Supplementary file 1 — The Case of Ms. Anthony.docxIntroducing ACEs Presentation.pptxSelf-Assessment.docx [file mep_2374-8265.10964-s001.zip › C. Self-Assessment.docx]

**ABehavioral Health Sciences Foundational Course: Adverse Childhood Experiences (ACEs)**

*For each item, please circle the number that best reflects your assessment of your degree of knowledge*

*(1 indicates limited to no knowledge and 5 reflects being comfortable with your degree of knowledge)*

Part I. Please complete BEFORE the session

|  | **Self-Assessment (Before Session)** | **Low High** | | | | |
| --- | --- | --- | --- | --- | --- | --- |
|  | I can define ACEs | 1 | 2 | 3 | 4 | 5 |
|  | I know what an ACE score is | 1 | 2 | 3 | 4 | 5 |
|  | I can differentiate between child abuse acts of commission and omission | 1 | 2 | 3 | 4 | 5 |
|  | I can identify 3 or more household dysfunctions classified as ACEs | 1 | 2 | 3 | 4 | 5 |
|  | I recognize the link between ACEs and adult mental health problems | 1 | 2 | 3 | 4 | 5 |
|  | I recognize the link between ACEs and chronic medical problems | 1 | 2 | 3 | 4 | 5 |
|  | I appreciate that neurotoxic stress can change brain functioning | 1 | 2 | 3 | 4 | 5 |
|  | I understand how high ACE scores are linked to a shorter life span | 1 | 2 | 3 | 4 | 5 |
|  | I can describe resilience | 1 | 2 | 3 | 4 | 5 |
|  | I recognize that ACEs can be prevented and treated | 1 | 2 | 3 | 4 | 5 |

Part II. Please complete AFTER the session

|  | **Self-Assessment (After Session)** | **Low High** | | | | |
| --- | --- | --- | --- | --- | --- | --- |
|  | I can define ACEs | 1 | 2 | 3 | 4 | 5 |
|  | I know what an ACE score is | 1 | 2 | 3 | 4 | 5 |
|  | I can differentiate between child abuse acts of commission and omission | 1 | 2 | 3 | 4 | 5 |
|  | I can identify 3 or more household dysfunctions categorized as ACEs | 1 | 2 | 3 | 4 | 5 |
|  | I recognize the link between ACEs and adult mental health problems | 1 | 2 | 3 | 4 | 5 |
|  | I recognize the link between ACEs and chronic medical problems | 1 | 2 | 3 | 4 | 5 |
|  | I appreciate that neurotoxic stress can change brain functioning | 1 | 2 | 3 | 4 | 5 |
|  | I understand how high ACE scores are linked to a shorter life span | 1 | 2 | 3 | 4 | 5 |
|  | I can describe resilience | 1 | 2 | 3 | 4 | 5 |
|  | I recognize that ACEs can be prevented and treated | 1 | 2 | 3 | 4 | 5 |

Please leave comments on the next page:

Comments: ……………………………………………………………………………………………………………………………………………………………………………………………………………………………………………………………………………………………………………………………………………………………………………………………………………………………………………………………………………………………………………………………………..

……………………………………………………………………………………………………………………………………………………………………………………………………………………………………………………………………………………………………………………………………………………………………………………………………………………………………………………………………………………………………………………………………..

……………………………………………………………………………………………………………………………………………………………………………………………………………………………………………………………………………………………………………………………………………………………………………………………………………………………………………………………………………………………………………………………………..
