# Supplementary material for: Introducing ACEs (Adverse Childhood Experiences) and Resilience to First-Year Medical Students
Source: MedEdPORTAL. 2020 Sep 15;16:10964. doi: 10.15766/mep_2374-8265.10964 (PMC7499813; doi:10.15766/mep_2374-8265.10964)
Supplement: Supplementary file 1 — The Case of Ms. Anthony.docxIntroducing ACEs Presentation.pptxSelf-Assessment.docx [file mep_2374-8265.10964-s001.zip › _Educational_Summary_Report_10964.pdf]

MedEdPORTAL® | The AAMC Journal of Teaching and Learning Resources

Original Publication | Open Access

# Introducing ACEs (Adverse Childhood Experiences) and Resilience to First-Year Medical Students

Edore Onigu-Otite, MD*, Sindhu Idicula, MD

*Corresponding author: Edore.Onigu-Otite@bcm.edu

## Abstract

**Introduction:** Adverse childhood experiences (ACEs) are associated with negative mental and physical health outcomes and predictive of higher sociodemographic risk. Introducing ACEs into undergraduate medical education is key to prevention, early recognition, and intervention. **Methods:** In a 1-hour lecture, held live and viewed online, we delivered a condensed introduction to ACEs to first-year medical students. Live-classroom participants completed pre-/postsession questionnaires self-assessing their knowledge of 10 content areas on a 5-point Likert scale. We analyzed quantitative data to determine mean scores and differences. We synthesized qualitative data obtained from feedback. **Results:** One hundred twenty-four students, including 32 live-classroom attendees and 92 online viewers, participated in this activity. Self-assessment scores increased in all content areas measured, with a mean increase of 1.5 ($p < .0001$). The most significant increases occurred in identifying household dysfunction as ACEs (increase of 2.3), calculating an ACE score (increase of 2.2), differentiating between child abuse acts of commission and omission (increase of 1.9), describing resilience (increase of 1.7), and recognizing the link between ACEs and chronic medical conditions (increase of 1.4). Participants found the lecture informative, appreciating the use of the case illustrating how ACEs impact health and an interactive slide on the risks conferred by cumulative ACEs. Learners welcomed the positive message of resilience. **Discussion:** Introducing ACEs in medical student education is feasible. Educating the next generation of health providers on ACEs while highlighting prevention and resilience and teaching trauma-informed care is crucial. This lecture can be readily incorporated into medical student curricula.

**Keywords:**
ACEs, Adverse Childhood Experiences, Child Maltreatment, Child Abuse, Mental Health, Chronic Medical Conditions, Trauma-Informed Care, Virtual Learning

## Educational Objectives

By the end of this session, learners will be able to:

1. Identify common adverse childhood experiences (ACEs).
2. Describe the longitudinal impact of ACEs on the mental and physical health of individuals.
3. List common risk factors and protective factors for child abuse and neglect.

## Introduction

Adverse childhood experiences (ACEs) are stressful events and experiences occurring before age 18. These include verbal, physical, or sexual abuse, as well as family dysfunction such as an incarcerated, mentally ill, or substance-abusing family member, witnessing a parent treated violently, or the absence of a parent because of divorce or separation.[1] In the early 1990s, a landmark study of about 17,000 adults, led by Felitti and colleagues, uncovered the association between ACEs and multiple medical and mental health conditions of public health concern.[2] This groundbreaking study found a robust association between the extent of exposure to abuse or household dysfunction during childhood and multiple risk factors for several of the leading causes of death in adults. Individuals with an ACE score of four or higher were 460% more likely to suffer from depression.[3] Having any ACE increased the risk of attempted suicide two- to fivefold throughout a person's life span.[4] Individuals who had six or more ACEs had over 24 times increased odds of attempting suicide.[4] This calls attention to the impact of ACEs on the burden of adult mental illness.

**Citation:**
Onigu-Otite E, Idicula S. Introducing ACEs (adverse childhood experiences) and resilience to first-year medical students. *MedEdPORTAL*. 2020;16:10964.
https://doi.org/10.15766/mep_2374-8265.10964

Copyright © 2020 Onigu-Otite and Idicula. This is an open-access publication distributed under the terms of the Creative Commons Attribution license.

More recent nationally representative studies have found a high burden of ACEs with about 60% of study participants reporting at least one ACE and about 25% reporting three or more ACEs.[5,6] ACEs are associated with leading causes of adult mortality and morbidity, including adult obesity,[7] sexually transmitted diseases,[8] diabetes, cancer,[9,10] and premature mortality.[11-14] ACEs are associated with other poor social outcomes, including impaired worker performance,[15] unemployment,[16] self-reported disability,[17] and a shortened life span.[13]

The Centers for Disease Control and Prevention (CDC) estimates the cost of child maltreatment to be $124 billion.[18] It has been suggested that a 10% reduction in ACE prevalence could equate to annual savings of 3 million disability-adjusted life-years or $105 billion.[19] ACEs have been linked to increased health service usage. Adults who report ACEs have higher household out-of-pocket medical costs. Adults reporting three or more ACEs are more likely to have medical expenses exceeding 10% of their household income and medical debt.[20] For every additional ACE score, the rate of the number of prescription drugs used increases by 62%.[21] There is a strong association between the number of ACEs experienced growing up (the ACE score) and the utilization of psychotropic medications in adulthood.[22] The substantial economic costs associated with the use of psychotropic medications, as well as management of side effects of these medications, and other costs of mental health treatment highlight the need to prevent, identify, and address ACEs early.

Economic research on human capital indicates significant cost savings potential may occur from the implementation of effective prevention strategies.[23] Data from the National Survey of Child and Adolescent Well-Being show that in children younger than 6, an ACE score of three or higher more than quadruples the risk of experiencing internalizing problems and almost quadruples the risk of experiencing either externalizing or total problems.[24] This highlights the need for early identification and intervention programs.

Protective factors within the context of ACEs in children have also been identified. These include growing up in a safe neighborhood, supportive neighbors, four or more neighborhood amenities, well-kept community, no household smoking, over five family meals per week, a parent who can talk to the child about things that matter and share ideas, and engagement in team sports.[25,26] Moreover, some prevention programs, such as preschool enrichment and early childhood home visitation programs, have shown successes, demonstrating 48%-52% reductions in rates of child abuse and neglect. Efforts that prevent ACEs could also potentially prevent adult chronic conditions, depression, health risk behaviors, and adverse socioeconomic outcomes.[6] Interestingly, researchers are increasingly investigating the development of resilience as a means to counter ACEs.[27] There is a call for the proactive promotion of positive childhood experiences for children within the context of ACEs to focus interventions on building strengths to promote well-being into adulthood.[28] Improving public health care requires a shift in focus to include the prevention of ACEs, resilience building, and trauma- or ACE-informed health care delivery.[29]

The trauma-informed approach, often referred to as trauma-informed care (TIC), includes an understanding of trauma and an awareness of the impact it can have across settings, services, and populations.[30] TIC impacts the health service industry with attendant health care costs. Yet, currently, there is a significant gap in medical education, with a relative scarcity of resources available on how to teach medical students about TIC. Closely related to this, ACEs have received comparatively little attention in formal medical education. In 2018, a study on a convenience sample of 20 University of California, Davis, medical students that was delivered in 2-hour modules over the course of 3 days concluded that trauma training can fill a knowledge gap and initial training can spark students' interest by demonstrating the relevance of trauma knowledge in medical practice.[31] In 2019, Pletcher, O'Connor, Swift-Taylor, and DallaPiazza developed a workshop on ACEs for medical students with an introduction to the protective effects of resilience and TIC,[32] which had a positive impact on medical students. In a study involving 18 second- and third-year pediatric residents, a flipped classroom model piloted during their developmental and behavioral pediatrics rotation found the most common practice change reported by residents was more systematically screening their patients for ACEs.[33] Schmitz, Light, Barry, and Hodges found that pediatric residents were not confident discussing ACEs, TIC, or resiliency even while acknowledging the importance of discussing ACEs, toxic stress, and resiliency with pediatric patients and their families, and highlighted the need for ACE education.[34] In this vein, the goal of our educational activity was to make a readily disseminatable version of an ACEs introduction, which could be easily implemented given limited curriculum time and educator availabilities. With the broad number of topics that need to be covered in undergraduate medication, the time allotted for new yet essential curriculum topics is limited.

Copyright © 2020 Onigu-Otite and Idicula. This is an open-access publication distributed under the terms of the Creative Commons Attribution license.

With this in mind, we adjusted our foundational behavioral sciences course to include an introduction to ACEs early in medical student training. Medical students were introduced to ACEs using a 1-hour lecture-style format. Lecture-style teaching is often considered a less effective method of developing skills, changing attitudes, or encouraging higher-order thinking. Nevertheless, under certain circumstances, it remains a viable means of transferring knowledge to large groups to increase awareness, providing core knowledge for student learning, and sparking interest in further study.[35] Although learners often prefer active learning, it is more feasible in smaller learner-group settings. For large groups of learners, particularly in the hundreds, lecture-style teaching is perhaps a more time-efficient and viable way of delivering large quantities of educational material.

This educational activity is unique as it has been designed to fit easily into current medical schedules and curricula in order to facilitate dissemination, including via online streaming, to students who, for various reasons, may not be physically present for the lecture. With this concise format, it can be more easily implemented, readily delivered to medical students, and potentially adapted to suit other learner groups. There is the option of modifying the activity to incorporate more active learning, such as a case-based discussion or a self-directed learning module. Furthermore, undergraduate medical education providers are increasingly looking to online options for lecture delivery that increase flexibility and accessibility and potentially expand dissemination of the educational material.[36] For the learner, this instructional activity provides in-classroom and online viewing options as well as further interactive online resources. For the instructor, this educational activity provides the necessary material and the space to be creative with an embedded active learning activity, based on the instructor's comfort level, knowledge, and experience with the content, with additional dissemination via online delivery.

In this learning activity, Felitti and colleagues' landmark study on ACEs, short videos, and infographics from the CDC on ACEs are used to provide baseline information on the public health implications of ACEs. An interactive chart that demonstrates the corresponding risks conferred by cumulative ACEs is included. The case of Ms. Anthony,[37] a competent professional referred by the chief of orthopedics to the chief of psychiatry, is used to illustrate the association between psychological trauma and the onset and progression of medical symptoms. The case demonstrates the challenges to treatment of her multiple chronic medical conditions, as well as the successes achieved in treatment upon addressing her ACEs.

## Methods

### Development

This lecture was created by the two course directors, both board-certified in child and adolescent psychiatry and general psychiatry. One course director was trained in psychoanalysis while the other was board-certified in addiction psychiatry and worked as the medical director of a center for child and family traumatic stress in an academic institution. We developed this educational activity for first-year medical students as part of the curriculum for the Behavioral Sciences Foundations course. The primary educational goal of this activity was to identify ACEs, link them to adult chronic medical conditions and negative health behaviors, and promote knowledge of resilience and intervention. A related goal was to create a teaching tool for educators that facilitated teaching ACEs and TIC during medical training. We designed the lecture to fit within the regular lecture schedule of our current undergraduate medical education curriculum. The lecture was designed to be given in a conference hall with both live-classroom attendance and live streaming to students not physically present. The curriculum change was approved by the curriculum committee of undergraduate medical student education at Baylor College of Medicine, Houston, Texas.

We designed the lecture for delivery within 1 hour, with the option of expanding by half an hour to include active learning via case-based discussion if time permitted. We obtained information on ACEs from multiple sources, including landmark articles, national population surveys and studies, and the CDC. An illustrative case connecting difficult-to-treat chronic medical conditions with ACEs was selected from the book *Fatal Pauses: Getting Unstuck Through the Power of No and the Power of Go*[37] (Appendix A). The lecture material consisted of a PowerPoint presentation, infographics, short videos, and a case-based presentation (Appendix B). An interactive chart from the CDC, illustrating the relationship between different ACE scores and the risk of developing health risk behaviors and physical or mental disease, was also included.[38]

We began the lecture with a case presentation, an introduction to the topic, a breakdown of the most prominent findings of the landmark study and relevant subsequent investigations, and a review of the case connecting the dots from the presenting medical conditions back to ACEs. Resilience, a protective factor against ACEs, was highlighted with examples using a prominent public figure. The lecture concluded with a short video from the CDC highlighting how ACEs can be prevented and addressed.

We constructed a self-assessment form (Appendix C) based on the lecture objectives and content. This form measured

Copyright © 2020 Onigu-Otite and Idicula. This is an open-access publication distributed under the terms of the Creative Commons Attribution license.

differences in student self-perception of knowledge in specific areas of importance presented in the lecture. The form consisted of 10 preselected content-specific areas assessed on a 5-point Likert scale where 1 reflected limited to no knowledge and 5 indicated being comfortable with one's degree of knowledge. This served not only to obtain data but also to provide the students with anchors and highlight areas to note within the condensed material.

Implementation
This lecture was treated no differently from most other lectures. Live attendance was not mandatory; students were not informed ahead of time that surveys would be taken. Slides were posted on Blackboard and made available to students 48 hours ahead of the lecture time in keeping with our institution's guidelines. Before the activity, self-assessment evaluation forms (Appendix C) were distributed to learners. Attendees completed pre- and postassessments. One faculty gave the lecture while another faculty facilitated. The lecture was recorded and available to students after the lecture.

Assessment
We obtained pre- and postsession scores, which were analyzed using Stata 15 (StataCorp). The mean differences in the pre- and postsession scores were determined, and a paired *t* test was used to determine statistical significance. Qualitative information was obtained from learner comments, and feedback was synthesized. Viewing stats were obtained from the audiovisual department for up to 30 days after the lecture was given.

## Results

We delivered the lecture in April 2019 during dedicated course time for first-year medical students. A total of 124 students attended or viewed the lecture. Ninety-two students viewed the lecture online, 33 on the same day and 59 within the next 30 days. Thirty-two live-classroom attendees completed the questionnaires. The mean presession score was 3.3 ($SD$ = 0.56) while the mean postsession score was 4.8 ($SD$ = 0.09), a mean difference of 1.5 ($SD$ = 0.51; 95% CI, 1.15-1.88; $p < .001$). Each item measured showed a mean increase in knowledge.

We found that baseline scores were lowest in the identification of household dysfunction as ACEs (2.3), knowledge of an ACE score (2.6), and differentiation between child abuse acts of omission and commission (2.8). At the end of the lecture, the highest scores were seen in the description of resilience (4.9), understanding how ACE score is associated with a shorter life span (4.8), the appreciation that neurotoxic stress changes brain function (4.8), definition of ACEs (4.8), and the calculation of an ACE score (4.8; see Figure).

The most considerable knowledge increase was seen in the identification of household dysfunction as ACEs (an increase of 2.3), calculating an ACE score (an increase of 2.2), differentiating between child abuse acts of commission and omission (an increase of 1.9), describing resilience (an increase of 1.7), and recognizing the link between ACEs and chronic medical conditions (an increase of 1.4). Comparatively, more knowledge increase was seen in the relationship between ACEs and chronic medical problems (an increase of 1.4) when compared to the recognition of the relationship between ACEs and adult mental health problems (an increase of 1.1).

Qualitative feedback showed that the sessions were well received (see Table). The learners enjoyed the lecture, identified various areas they found particularly helpful plus a problem area, and gave suggestions for improvement.

## Discussion

This lecture fills a gap in medical curriculum education, specifically by increasing awareness of ACEs and how they relate to medical and mental health conditions, including challenges to care. The ability to deliver this condensed lecture within 1 hour, with the achieved outcome of increased self-perception of knowledge, demonstrates that a comprehensive awareness of ACEs can be introduced in a time-efficient manner early in medical training. Including a case that synthesized ACEs, chronic medical conditions, and response to treatment was high yield, was viewed as useful, and generated interest among attendees. That the initial patient referral came from surgery to psychiatry is worthy of note. Increasing awareness of the need for mental health treatment within difficult-to-treat chronic medical diseases of public health concern could also be a target of education for the young medical student. It has been suggested that medical practitioners should consider ACEs in difficult-to-treat cases where otherwise effective remedies have not been sufficient.[39]

At baseline, identifying household dysfunction as an ACE received a low score. While child abuse and neglect are more clearly defined as harmful events, dysfunctional home environments often go undetected as a harmful factor detrimental to child and, later, adult development. Interestingly, at the end of the lecture, the recognition of the link between ACEs and chronic medical problems was higher than the recognition of the link between ACEs and adult mental health problems. This indicates that this group of learners assimilated more knowledge on the association of ACEs with medical diseases than with

Copyright © 2020 Onigu-Otite and Idicula. This is an open-access publication distributed under the terms of the Creative Commons Attribution license.

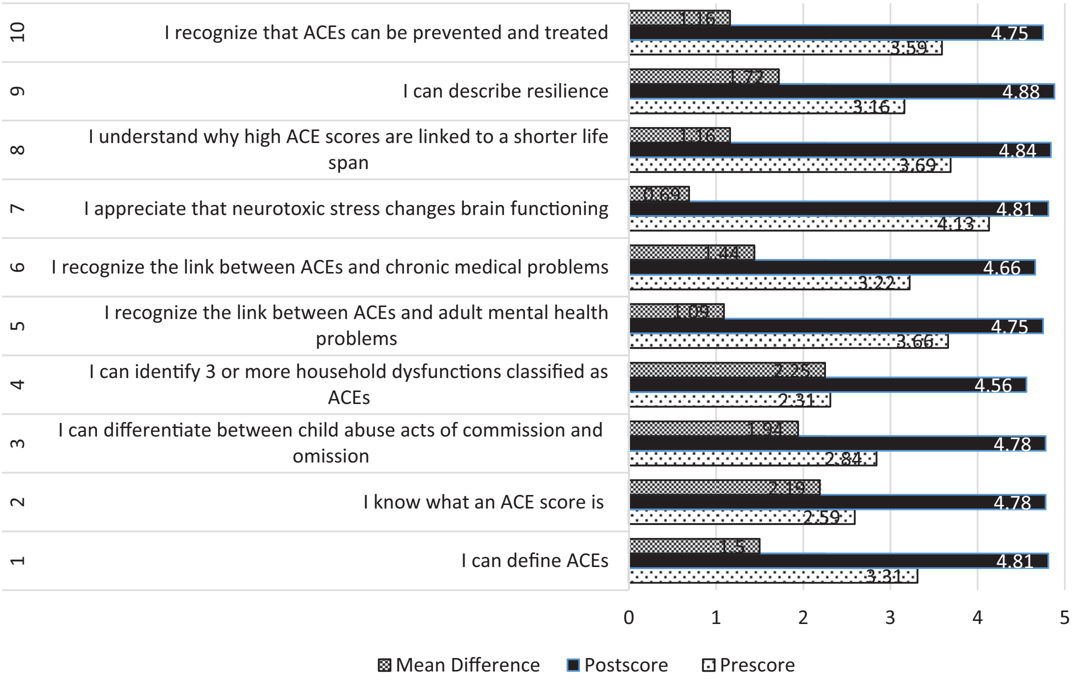


**Figure.** Mean pre- and postsession scores and difference. Abbreviation: ACE, adverse childhood experience.

mental health disorders. Perhaps it shows an increased aptitude in young medical trainees for understanding the causation of medical illness as multifaceted and inclusive of ecological experiences. Notably, at baseline, the recognition of the link between ACEs and adult mental health was comparatively higher starting out, which may indicate that it is more intuitive knowledge in general. The appreciation that neurotoxic stress changes brain functioning was relatively high at baseline. This may have been due in part to the overlap between neurology and behavioral health sciences lectures that were given in the same term.

**Table.** Summary of Qualitative Feedback

| Quality | Comment |
|---|---|
| Utility of information | "Informative lecture about ACEs."<br>"A good reminder about how so much of a child's future is shaped by their family and environment."<br>"Resilience sent a positive message about what we can do about ACEs."<br>"Found today's lecture especially poignant as it is the aspect of behavioral sciences I am most interested in." |
| Utility of teaching tools | "Liked the use of the example video at the beginning and videos throughout."<br>"The case audio was helpful in setting how ACES result in adult behaviors that physicians may not recognize as due to early trauma."<br>"The interactive slide was really helpful for seeing the outcomes." |
| Suggestions for future implementation | "Not sure all the videos were necessary as parts of the lecture seemed a bit rushed."<br>"Maybe get a better reading of the case presentation—the artificial intelligence reader was a little hard to understand. I would rather have simply heard about the case from the instructors."<br>"Maybe talking about how physicians can recognize or ask about these factors would be an interesting addition." |

Abbreviation: ACEs, adverse childhood experiences.

The qualitative feedback highlighted the students' interest in learning more about how physicians can identify patients with ACEs. Using the ACE score as a screener may be helpful in clinical practice. However, it is important to note that there are limitations to using original ACEs as a screening tool as they stop at individual and household ACEs while children and individuals can be adversely affected by bullying, school and community violence, and natural disasters.[40] Nevertheless, ACE-based screenings and referrals are becoming increasingly used in adult and pediatric primary care.[41,42]

A positive outcome to highlight is that learners' ability to describe resilience, a protective factor in an individual's development, increased. This is important because significant attention is paid to the adversity associated with ACEs, but the strengths and resilience in individuals with ACEs, which enable them to overcome and succeed in life, are often overlooked. It can be anticipated that learners may potentially look for and highlight

Copyright © 2020 Onigu-Otite and Idicula. This is an open-access publication distributed under the terms of the Creative Commons Attribution license. 

strengths in individuals with ACEs, which may bolster belief and further enable such individuals to overcome the negative start in life associated with ACEs. This may help reduce any sense of provider helplessness when encountering ACEs in practice.

The increasing use of e-learning in medical education now includes the trauma field. For example, in a recent study, Schmitz, Light, Barry, and Hodges created an online module for pediatricians.[34] In the case of the present study, the two modes of delivery of the activity and comparative attendance are worthy of mention. About one-quarter of the students attended the live classroom session, while three-quarters of the class viewed the lecture online. The addition of the online mode of lecture delivery thus tripled the participation and significantly boosted the dissemination of this instructional material.

Finally, there is a relative lack of tools and resources available to teach medical students about ACEs. This educational activity adds to the existing literature by providing a readily implementable educational activity that is well suited to fit within the current structure of medical education, with the additional benefit of inclusion of active learning via a case-based discussion.

### Limitations

A limitation of this study was that a relatively low number of students attended the live classroom session and were therefore available to complete the questionnaires. The majority of students who participated did so online. Questionnaires were not obtained from online viewers, which would have provided additional information. While it would have been helpful to have the same questionnaire provided to the online viewers, assuring anonymity to students would have been difficult given that students must log in with their unique student identities to view course lectures. The questionnaires measured students' perception of knowledge as opposed to actual knowledge, which would have required more rigorous data collection. The students did not receive debriefing after the lecture, which is important because students may have experienced ACEs of their own and could potentially have benefitted from debriefing.

A significant challenge we faced was distilling the large body of ACEs material into just 50 minutes of lecture time; necessary compromises had to be made. This applied to the decision to have the illustrative case read by artificial intelligence, which might have reduced the case's efficacy for this lecture. This could be resolved if the case were shortened and read by a live person or facilitator. We think reading the case from a script would be better for capturing the details and process of discovery and treatment related to ACEs as contained in the original story.[37] However, the case is utilized more operationally later in the lecture in a way that connects the dots between difficult-to-treat medical problems and ACEs. Although closely related, a key topic to expand on in this lecture is the concept of TIC, which would be important to include if more time is available.

A relative limitation was the use of lecture-style teaching, which encourages passive learning. Certainly, incorporating more active learning activities into this lecture is desirable if additional time is available. For future implementation, we suggest expanding slides 29 and 30 into an active learning activity where the large group can break up into smaller groups for the case discussion. Students can be asked to track ACEs to mental health and medical problems (as depicted in slide 29). To guide this, we suggest asking learners to do the following:

1. Discuss ACEs present in the case.
2. Discuss the mental health symptoms/problems.
3. Discuss factors indicative of resilience.
4. Discuss the negative health behaviors (physical and/or mental).
5. Discuss the chronic medical disease conditions and potential challenges to treatment.

Similarly, students can be asked to connect the dots in the case and track mental health treatment to wellness, including mental health and physical health (as depicted in slide 30). To guide this, we suggest asking learners to do the following:

1. Discuss health care usage factors.
2. Discuss loss of productivity.
3. Discuss the impact of ACEs on adult life in this case.
4. Discuss access and barriers to treatment for mental health care in this case.
5. Discuss factors related to successful treatment in this case.

These are broad questions that could generate a lively discussion. This expansion, whenever feasible, could incorporate a case-based active learning piece that provides the students with the space to process, critically appraise, and apply the new knowledge learned. To do this effectively, additional facilitators may be needed depending on the size of the group.

### Conclusion

Creating this lecture was important because it provides a tool to teach ACEs to medical students and introduces the trauma-informed approach, an essential component of TIC in health institutions. This is an educational curriculum intervention intended to introduce the knowledge of ACEs at the early

Copyright © 2020 Onigu-Otite and Idicula. This is an open-access publication distributed under the terms of the Creative Commons Attribution license.

stages of medical education. Identifying child abuse, including physical, sexual, and emotional acts, is a straightforward concept that is generally known and accepted in society. However, household dysfunction is often overlooked as a source of neurotoxic stress for the growing child. With this educational intervention, students' knowledge increased the most in their ability to identify household dysfunctions as adverse to the development of the child and therefore worthy of attention and intervention. This educational activity promoted increases in students' self-perception of knowledge and reflected student satisfaction. It is expected that as medical students go through their careers, they can build on this foundation and develop into more trauma-sensitive medical personnel along the different paths their medical careers lead. The lecture is deliverable within current medical education structures, with the online version increasing flexibility and accessibility for students and consequently boosting attendance.

## Appendices

A. The Case of Ms. Anthony.docx

B. Introducing ACEs Presentation.pptx

C. Self-Assessment.docx

*All appendices are peer reviewed as integral parts of the Original Publication.*

**Edore Onigu-Otite, MD:** Associate Professor, Menninger Department of Psychiatry and Behavioral Sciences, Baylor College of Medicine; Associate Course Director, Behavioral Sciences Foundations Course, School of Medicine, Baylor College of Medicine

**Sindhu Idicula, MD:** Assistant Professor, Menninger Department of Psychiatry and Behavioral Sciences, Baylor College of Medicine; Course Director, Behavioral Sciences Foundations Course, School of Medicine, Baylor College of Medicine

### Acknowledgments
We would like to thank Nadia Ismail, MD, MPH, MEd, the Associate Dean of Curriculum, and Jennifer Christner, MD, the Dean, School of Medicine, Baylor College of Medicine. We especially thank Stuart Yudofsky, Distinguished Professor Emeritus and former Chair of Psychiatry; Wayne Goodman, MD, D. C. and Irene Ellwood Chair of Psychiatry; Asim Shah, MD, Vice-Chair of Psychiatry; and Kim-Lan T. Czelusta, MD, Associate Professor and Vice-Chair for Education, Menninger Department of Psychiatry and Behavioral Sciences, Baylor College of Medicine, Houston, Texas. Most importantly, we thank each of the medical students who participated in this activity.

### Disclosures
None to report.

### Funding/Support
None to report.

### Ethical Approval
Reported as not applicable.

## References

1. Adverse childhood experiences reported by adults—five states, 2009. *MMWR Morb Mortal Wkly Rep*. 2010;59(49):1609-1613.
2. Felitti VJ, Anda RF, Nordenberg D, et al. Relationship of childhood abuse and household dysfunction to many of the leading causes of death in adults: the Adverse Childhood Experiences (ACES) Study. *Am J Prev Med*. 1998;14(4):245-258. https://doi.org/10.1016/S0749-3797(98)00017-8
3. Chapman DP, Whitfield CL, Felitti VJ, Dube SR, Edwards VJ, Anda RF. Adverse childhood experiences and the risk of depressive disorders in adulthood. *J Affect Disord*. 2004;82(2):217-225. https://doi.org/10.1016/j.jad.2003.12.013
4. Dube SR, Anda RF, Felitti VJ, Chapman DP, Williamson DF, Giles WH. Childhood abuse, household dysfunction, and the risk of attempted suicide throughout the life span: findings from the Adverse Childhood Experiences Study. *JAMA*. 2001;286(24): 3089-3096. https://doi.org/10.1001/jama.286.24.3089
5. Merrick MT, Ford DC, Ports KA, Guinn AS. Prevalence of adverse childhood experiences from the 2011-2014 Behavioral Risk Factor Surveillance System in 23 states. *JAMA Pediatr*. 2018; 172(11):1038-1044. https://doi.org/10.1001/jamapediatrics.2018.2537
6. Merrick MT, Ford DC, Ports KA, et al. Vital signs: estimated proportion of adult health problems attributable to adverse childhood experiences and implications for prevention—25 states, 2015–2017. *MMWR Morb Mortal Wkly Rep*. 2019;68(44): 999-1005. https://doi.org/10.15585/mmwr.mm6844e1
7. Williamson DF, Thompson TJ, Anda RF, Dietz WH, Felitti V. Body weight and obesity in adults and self-reported abuse in childhood. *Int J Obes (Lond)*. 2002;26(8):1075-1082. https://doi.org/10.1038/sj.ijo.0802038
8. Hillis SD, Anda RF, Felitti VJ, Nordenberg D, Marchbanks PA. Adverse childhood experiences and sexually transmitted diseases in men and women: a retrospective study. *Pediatrics*. 2000;106(1):E11. https://doi.org/10.1542/peds.106.1.e11
9. Ports KA, Holman DM, Guinn AS, et al. Adverse childhood experiences and the presence of cancer risk factors in adulthood: a scoping review of the literature from 2005 to 2015. *J Pediatr Nurs*. 2019;44:81-96. https://doi.org/10.1016/j.pedn.2018.10.009
10. Holman DM, Ports KA, Buchanan ND, et al. The association between adverse childhood experiences and risk of cancer in

Copyright © 2020 Onigu-Otite and Idicula. This is an open-access publication distributed under the terms of the Creative Commons Attribution license.

adulthood: a systematic review of the literature. *Pediatrics*. 2016;138(suppl 1):S81-S91. https://doi.org/10.1542/peds.2015-4268L

11. Brown DW, Anda RF, Felitti VJ, et al. Adverse childhood experiences are associated with the risk of lung cancer: a prospective cohort study. *BMC Public Health*. 2010;10:20. https://doi.org/10.1186/1471-2458-10-20
12. Anda R, Tietjen G, Schulman E, Felitti V, Croft J. Adverse childhood experiences and frequent headaches in adults. *Headache*. 2010;50(9):1473-1481. https://doi.org/10.1111/j.1526-4610.2010.01756.x
13. Brown DW, Anda RF, Tiemeier H, et al. Adverse childhood experiences and the risk of premature mortality. *Am J Prev Med*. 2009;37(5):389-396. https://doi.org/10.1016/j.amepre.2009.06.021
14. Patterson ML, Moniruzzaman A, Somers JM. Setting the stage for chronic health problems: cumulative childhood adversity among homeless adults with mental illness in Vancouver, British Columbia. *BMC Public Health*. 2014;14:350. https://doi.org/10.1186/1471-2458-14-350
15. Anda RF, Felitti VJ, Fleisher VI, et al. Childhood abuse, household dysfunction, and indicators of impaired adult worker performance. *Perm J*. 2004;8(1):30-38. https://doi.org/10.7812/TPP/03-089
16. Liu Y, Croft JB, Chapman DP, et al. Relationship between adverse childhood experiences and unemployment among adults from five US states. *Soc Psychiatry Psychiatr Epidemiol*. 2013;48(3):357-369. https://doi.org/10.1007/s00127-012-0554-1
17. Schüssler-Fiorenza Rose SM, Xie D, Stineman M. Adverse childhood experiences and disability in U.S. adults. *PM R*. 2014;6(8):670-680. https://doi.org/10.1016/j.pmrj.2014.01.013
18. Fang X, Brown DS, Florence CS, Mercy JA. The economic burden of child maltreatment in the United States and implications for prevention. *Child Abuse Negl*. 2012;36(2):156-165. https://doi.org/10.1016/j.chiabu.2011.10.006
19. Bellis MA, Hughes K, Ford K, Ramos Rodriguez G, Sethi D, Passmore J. Life course health consequences and associated annual costs of adverse childhood experiences across Europe and North America: a systematic review and meta-analysis. *Lancet Public Health*. 2019;4(10):e517-e528. https://doi.org/10.1016/S2468-2667(19)30145-8
20. Schickedanz AB, Escarce JJ, Halfon N, Sastry N, Chung PJ. Adverse childhood experiences and household out-of-pocket healthcare costs. *Am J Prev Med*. 2019;56(5):698-707. https://doi.org/10.1016/j.amepre.2018.11.019
21. Anda RF, Brown DW, Felitti VJ, Dube SR, Giles WH. Adverse childhood experiences and prescription drug use in a cohort study of adult HMO patients. *BMC Public Health*. 2008;8:198. https://doi.org/10.1186/1471-2458-8-198
22. Anda RF, Brown DW, Felitti VJ, Bremner JD, Dube SR, Giles WH. Adverse childhood experiences and prescribed psychotropic medications in adults. *Am J Prev Med*. 2007;32(5):389-394. https://doi.org/10.1016/j.amepre.2007.01.005
23. Larkin H, Felitti VJ, Anda RF. Social work and adverse childhood experiences research: implications for practice and health policy. *Soc Work Public Health*. 2014;29(1):1-16. https://doi.org/10.1080/19371918.2011.619433
24. Clarkson Freeman PA. Prevalence and relationship between adverse childhood experiences and child behavior among young children. *Infant Ment Health J*. 2014;35(6):544-554. https://doi.org/10.1002/imhj.21460
25. Robles A, Gjelsvik A, Hirway P, Vivier PM, High P. Adverse childhood experiences and protective factors with school engagement. *Pediatrics*. 2019;144(2):e20182945. https://doi.org/10.1542/peds.2018-2945
26. Easterlin MC, Chung PJ, Leng M, Dudovitz R. Association of team sports participation with long-term mental health outcomes among individuals exposed to adverse childhood experiences. *JAMA Pediatr*. 2019;173(7):681-688. https://doi.org/10.1001/jamapediatrics.2019.1212
27. Merrick JS, Narayan AJ, DePasquale CE, Masten AS. Benevolent childhood experiences (BCEs) in homeless parents: a validation and replication study. *J Fam Psychol*. 2019;33(4):493-498. https://doi.org/10.1037/fam0000521
28. Bethell C, Jones J, Gombojav N, Linkenbach J, Sege R. Positive childhood experiences and adult mental and relational health in a statewide sample: associations across adverse childhood experiences levels. *JAMA Pediatr*. 2019;173(11):e193007. https://doi.org/10.1001/jamapediatrics.2019.3007
29. Hughes K, Bellis MA, Hardcastle KA, et al. The effect of multiple adverse childhood experiences on health: a systematic review and meta-analysis. *Lancet Public Health*. 2017;2(8):e356-e366. https://doi.org/10.1016/S2468-2667(17)30118-4
30. Substance Abuse and Mental Health Services Administration. *Trauma-Informed Care in Behavioral Health Services*. Substance Abuse and Mental Health Services Administration; 2014. Treatment Improvement Protocol (TIP) Series 57. HHS publication no. (SMA) 13-4801.
31. Goldstein E, Murray-García J, Sciolla AF, Topitzes J. Medical students' perspectives on trauma-informed care training. *Perm J*. 2018;22:17-126. https://doi.org/10.7812/TPP/17-126
32. Pletcher BA, O'Connor M, Swift-Taylor ME, DallaPiazza M. Adverse childhood experiences: a case-based workshop introducing medical students to trauma-informed care. *MedEdPORTAL*. 2019;15:10803. https://doi.org/10.15766/mep_2374-8265.10803
33. Stefanski K, Mason K. Acing education: pilot curriculum on adverse childhood experiences. *Med Educ*. 2017;51(11):1167-1168. https://doi.org/10.1111/medu.13436
34. Schmitz A, Light S, Barry C, Hodges K. Adverse childhood experiences and trauma-informed care: an online module for

Copyright © 2020 Onigu-Otite and Idicula. This is an open-access publication distributed under the terms of the Creative Commons Attribution license.

pediatricians. *MedEdPORTAL*. 2019;15:10851. https://doi.org/10.15766/mep_2374-8265.10851

35. Cantillon P. Teaching large groups. *BMJ*. 2003;326:437. https://doi.org/10.1136/bmj.326.7386.437
36. Warren N, Parker S, Khoo T, Cabral S, Turner J. Challenges and solutions when developing online interactive psychiatric education. *Australas Psychiatry*. 2020;28(3):359-362. https://doi.org/10.1177/1039856220901477
37. Yudofsky SC. *Fatal Pauses: Getting Unstuck Through the Power of No and the Power of Go*. American Psychiatric Publishing; 2015.
38. Adverse childhood experiences: looking at how ACES affect our lives & society. Centers for Disease Control and Prevention. Updated February 22, 2013. https://vetoviolence.cdc.gov/apps/phl/resource_center_infographic.html
39. Edwards VJ, Anda RF, Gu D, Dube SR, Felitti VJ. Adverse childhood experiences and smoking persistence in adults with smoking-related symptoms and illness. *Perm J*. 2007;11(2):5-13. https://doi.org/10.7812/TPP/06-110
40. Stein S, Swerlick A, Chokshi B. Employing an adaptive leadership framework to childhood adversity screening. *Pediatrics*. 2020; 145(1):e20183820. https://doi.org/10.1542/peds.2018-3820
41. Choi KR, McCreary M, Ford JD, Rahmanian Koushkaki S, Kenan KN, Zima BT. Validation of the Traumatic Events Screening Inventory for ACEs. *Pediatrics*. 2019;143(4):e20182546. https://doi.org/10.1542/peds.2018-2546
42. Van Niel C, Pachter LM, Jr Wade R, Felitti VJ, Stein MT. Adverse events in children: predictors of adult physical and mental conditions. *J Dev Behav Pediatr*. 2014;35(8):549-551. https://doi.org/10.1097/DBP.0000000000000102

**Received:** November 24, 2019
**Accepted:** March 9, 2020
**Published:** September 15, 2020

Copyright © 2020 Onigu-Otite and Idicula. This is an open-access publication distributed under the terms of the Creative Commons Attribution license.
